# A Novel Probe for Spliceosomal Proteins that Induces Autophagy and Death of Melanoma Cells Reveals New Targets for Melanoma Drug Discovery

**DOI:** 10.33594/000000164

**Published:** 2019

**Authors:** Manikandan Palrasu, Anna M. Knapinska, Juan Diez, Lyndsay Smith, Travis LaVoi, Marc Giulianotti, Richard A. Houghten, Gregg B. Fields, Dmitriy Minond

**Affiliations:** a Department of Drug Discovery and Development, Harrison School of Pharmacy, Auburn University, Auburn, AL, USA,; b Department of Chemistry & Biochemistry, Center for Molecular Biology & Biotechnology, Florida Atlantic University, Jupiter, FL, USA,; c Rumbaugh-Goodwin Institute for Cancer Research, Nova Southeastern University, Fort Lauderdale, FL, USA,; d Torrey Pines Institute for Molecular Studies, Port St. Lucie, FL, USA,; e Dr. Kiran C. Patel College of Allopathic Medicine, Nova Southeastern University, Fort Lauderdale, FL, USA

**Keywords:** Mechanism of action, Autophagy, Spliceosomal protein binding, Target identification, Melanoma

## Abstract

**Background/Aims:**

Despite recent advances in melanoma drug discovery, the average overall survival of patients with late stage metastatic melanoma is approximately 3 years, suggesting a need for approaches that identify new melanoma targets. We have previously reported a discovery of novel anti-melanoma compound 2155–14 (Onwuha-Ekpete et al., J Med Chem. 2014 Feb 27; 57(4):1599–608). In the report presented herein we aim to identify its target(s) and mechanism of action.

**Methods:**

We utilized biotinylated analog of 2155–14 to pull down its targets from melanoma cells. Proteomics in combination with western blot were used to identify the targets. Mechanism of action of 2155–14 was determined using flow cytometry, RT-PCR, microscopy, western blot, and enzymatic activity assays. Where applicable, one-way analysis of variance (ANOVA) was used followed by Dunnett post hoc test.

**Results:**

In the present study, we identified ATP-dependent RNA helicase DDX1 and heterogeneous nuclear ribonucleoproteins (hnRNPs) H1, H2 and A2/B1 as targets of anti-melanoma compound 2155–14. To the best of our knowledge, this is a first report suggesting that these proteins could be targeted for melanoma therapy. Mechanistic investigations showed that 2155–14 induces ER stress leading to potentiation of basal autophagy resulting in melanoma cell death in BRAF and NRAS mutated melanoma cells.

**Conclusion:**

Identification of mode of action of 2155–14 may provide insight into novel therapies against a broad range of melanoma subtypes. These studies were enabled by the novel probe derived from a mixture-based library, an important class of chemical biology tools for discovering novel targets.

## Introduction

As estimated by the National Cancer Institute, there are more than 900, 000 people living with melanoma in the USA [1]. The estimated number of new cases in 2018 was more than 178, 000 in US and 300, 000 globally [2] with the number of deaths in US more than 10, 000 [3] and more than 60, 000 worldwide [4]. If melanoma is resected before it metastasizes, the 5-year survival rate is 98%. However, if allowed to metastasize, the 5-year survival rate is only 10–15%. New therapies and approaches are urgently needed, and melanoma is one of the most active areas of oncological drug discovery with multiple active clinical trials [5].

There are several melanoma subtypes based on the molecular alterations present [6]. Melanoma proliferation is mainly regulated by the *Ras/Raf/MEK/ERK* pathway. Most of the identified molecular alterations (i.e. mutations, deletions, and amplifications) that drive melanoma are concentrated in this pathway. *ERK* is hyperactivated in approximately 90% of human melanomas [7]. *NRAS* gain-of-function mutation Q61L occurs in 15–30% of cases [8]. *BRAF* is mutated in 50–70% of melanomas [8]. In most melanoma cases, more than one alteration is present, which could necessitate different therapeutic approaches.

An almost inevitable acquired resistance to therapy is another hallmark of melanoma. Chemo (dacarbazine, temolozomide), immuno (IL-2, ipilimumab), and targeted (vemurafenib, dabrafenib, trametinib, cobimetinib) monotherapies usually result in resistance [9] which necessitates combination therapies using the aforementioned drugs. In January 2014, the FDA approved a BRAF/MEK inhibitor combination (dabrafenib/trametinib) for BRAF-mutant metastatic melanoma [10], which demonstrated higher response rates (76% versus 59%) and slightly longer median progression-free survival (PFS) than dabrafenib or vemurafenib monotherapies (9.4 versus 6.9 months) with less toxicity. Some toxicity was reported, however, such that >50% of patients had to reduce the dosage and 9% discontinued the treatment. The resistance to this drug combination has already been reported [11–13]. Most recently, the FDA approved the BRAF/MEK inhibitor combination vemurafenib/cobimetinib. Overall survival (OS) in phase III trials was 25–26 months for dabrafenib/trametinib and 22 months for vemurafenib/cobimetinib [11].

Monotherapy using selective CDK 4/6 inhibitors (e.g., palbociclib, ribocicllib, abemaciclib) has shown a limited response (~3% response rate) in melanoma clinical trials [14]. CDK 4/6 inhibitors are currently being evaluated in combinations with BRAF and MEK inhibitors against BRAF- and NRAS-mutated melanomas. Combination of PD-1 and CTLA-4 immunological checkpoint inhibitors nivolumab (marketed as Opdivo) and ipilimumab [15, 16] exhibited overall response rate, PFS, and OS similar to dabrafenib/trametinib, but with a longer lasting effect after termination of therapy, likely due to the induced monitoring of cancer cells by immune cells.

Despite recent advances in melanoma drug discovery, the average overall survival of patients with late stage metastatic melanoma is ~3 years. Instances of complete response are very rare; therefore, more life-prolonging therapies are needed. This suggests a need for new approaches and targets for melanoma drug discovery.

In the studies presented herein we utilized a chemical probe based on the compounds we previously described [17] to study the mechanism of action and molecular targets in melanoma. Our results suggest a potential for novel targets for melanoma therapy that are common amongst different melanoma molecular subtypes [6].

## Materials and Methods

### General synthesis procedure for pyrrolidine-bis-diketopiperazines and tagged analogs (Supplementary Fig. 1)

For all supplemental material see www.cellphysiolbiochem.com.

All compounds were synthesized *via* solid-phase methodology on 4-methylbenzhydrylamine hydrochloride resin (MBHA) (1.2 mmol/g, 100–200 mesh) using the “tea-bag” approach [18] with some modifications to the method previously described elsewhere [19]. Boc- and Fmoc-amino acids (6 equiv) were coupled utilizing standard coupling procedures with hydroxybenzotriazole hydrate (HOBt, 6 equiv) and N,N’-diisopropylcarbodiimide (DIC, 6 equiv) in dimethylformamide (DMF, 0.1 M) for 120 min. Boc protecting groups were removed with 55% trifluoroacetic acid (TFA)/45% dichloromethane (DCM) (1x, 30 min) and subsequently neutralized with 5% diisopropylethylamine (DIEA)/95% DCM (3x, 2 min). Fmoc protecting groups were removed with 20% piperidine/80% DMF (2x, 30 min). Carboxylic acids were coupled utilizing standard coupling procedures (10 equiv) with HOBt (10 equiv) and DIC (10 equiv) in DMF (0.1 M) for 120 min. Trityl protection was carried out by neutralizing bags with 5% DIEA/95% DCM (3x, 2min), treating bags with triphenylmethyl chloride (Trt-Cl, 5 equiv) and DIEA (10 equiv) in 10%DMF/90%DCM (0.1 M) for 2 h, neutralizing bags again (3x, 2 min), and repeating Trt-Cl treatment (18 h). The trityl group was removed by washing with a solution of 2% TFA/5% triisopropylsilane (TIS)/93% DCM (3x, 2 min). Biotin (10 equiv) was coupled utilizing standard coupling procedures with DIC (10 equiv) in DMF (0.1 M) for 120 min. Dansyl chloride (10 equiv) was acylated utilizing DIEA (10 equiv) in DMF (0.1 M) for 120 min. Completion of all couplings was monitored with a ninhydrin test. Compounds were reduced to polyamines using a 40x excess of borane (1.0 M in tetrahydrofuran (THF)) over each amide bond in a glass vessel under nitrogen at 65 °C for 72 h. The solution was then poured off, the reaction was quenched with methanol (MeOH), and the bags were washed with THF (1x, 1 min) and MeOH (4x, 1 min) and allowed to air dry. Once dry, the bags were treated with piperidine overnight at 65 °C in a glass vessel. The solution was poured off, and the bags were washed with DMF (2x, 1 min), DCM (2x, 1 min), MeOH (1x, 1 min), DMF (2x, 1 min), DCM (2x, 1 min), and MeOH (1x, 1 min), and allowed to air dry. Completion of reduction was checked by cleaving a control sample and analyzing using LC-MS. As previously reported by our group and others the reduction of polyamides with borane is free of racemization [20–22]. Diketopiperazine cyclization was performed under anhydrous conditions (<22% humidity). The dry bags were washed with anhydrous DMF (2x, 1 min), added to a solution of 1, 1’-oxalyldiimidazole (5 fold excess for each cyclization site) in anhydrous DMF (0.1 M), and shaken at room temperature overnight. The solution was poured off and the bags were rinsed with DMF (3x, 1 min) and DCM (3x, 1 min). Completion of cyclization was checked by cleaving a control sample and analyzing by LC-MS. The compounds were then cleaved from the resin with hydrofluoric acid (HF) in the presence of anisole in an ice bath at 0 °C for 90 min and extracted using 95% acetic acid (AcOH)/5% H_2_O (2x, 5 mL). Final crude products were purified using HPLC as described below. All chirality was generated from the corresponding amino acids.

### Compound purification and characterization

The final compounds were purified using preparative HPLC with a dual pump Shimadzu LC-20AB system equipped with a Luna C18 preparative column (21.5 × 150 mm, 5 micron) at λ = 214 nm, with a mobile phase of (A) H_2_O (+0.1% formic acid) and (B) acetonitrile (ACN) (+0.1% formic acid) at a flow rate of 13 mL/min, where the gradient was varied by compound based on hydrophobicity. ^1^H NMR and ^13^C NMR spectra were recorded in DMSO-d6 on a Bruker Ascend 400 MHz spectrometer at 400.14 and 100.62 MHz, respectively, and MALDI-TOF mass spectra were recorded using an Applied Biosystems Voyager DE-PRO Biospectrometry workstation. The purities of synthesized compounds were confirmed to be greater than 95% by liquid chromatography and mass spectrometry on a Shimadzu LCMS-2010 instrument with ESI Mass Spec and SPD-20A Liquid Chromatograph with a mobile phase of (A) H_2_O (+0.1% formic acid) and (B) ACN (+0.1% formic acid) (gradient of 5–95% B over 6 min with a 4 min rinse).

### Cell viability assays

Briefly, cells were plated in 384-well plates in 8 μL of media. Test compounds and dabrafenib (pharmacological assay control) were prepared as 10-point, 1:3 serial dilutions starting at 300 μM, then added to the cells (4 μL per well) using the Biomek NX^P^. Plates were incubated for 72 h at 37 °C, 5% CO_2_, and 95% relative humidity. After incubation, 4 μL of CellTiter-Glo® (Promega cat#: G7570) were added to each well, and incubated for 15 min at room temperature. Luminescence was recorded using a Biotek Synergy H4 multimode microplate reader. Viability was expressed as a percentage relative to wells containing media only (0%) and wells containing cells treated with DMSO only (100%). Three parameters were calculated on a per-plate basis: (a) the signal-to-background ratio (S/B); (b) the coefficient for variation [CV; CV = (standard deviation/mean) × 100)] for all compound test wells; and (c) the Z’-factor (18). The IC_50_ value of the pharmacological control (dabrafenib, LC Laboratories # G-4408) was also calculated to ascertain the assay robustness.

In case of viability rescue assays, cells were pre-treated with caspase, calpain, and autophagy inhibitors for 1–3 h before addition of test compounds. Time course viability assay was done with luminescence measurements performed at 4, 24, 48, and 72 h.

### Luciferase counterscreen assay

Lead compounds and various inhibitors used in present study were tested for inhibition of luciferase from the CellTiter-Glo® assay kit (Promega cat#: G7570). The ATP concentration in the luciferase assay was matched to the response produced by WM266–4 cells. Test compounds were prepared as 10-point, 1:3 serial dilutions starting at 300 μM, then added to the DMEM (5 μL per well) using the Biomek NX^P^. Plates were incubated for 1 h at 37°C, 5% CO_2_ and 95% relative humidity. After incubation, 5 μL of CellTiter-Glo® (Promega cat#: G7570) was added to each well, and incubation continued for 15 min at room temperature. Luminescence was recorded using a Biotek Synergy H4 multimode microplate reader. Inhibition was expressed as a percentage relative to wells containing media only (0%) and wells containing CellTiter-Glo® (100%).

### MAPK-Akt flow cytometry assay

WM266–4 cells were seeded at 250, 000 cells/well in E-MEM medium supplemented with 10% FBS and 1% penicillin/streptomycin in 6 well plates (Greiner Bio-One CellStar cat# 655180) and allowed to adhere overnight. After incubation cells were treated with control (25 μM dabrafenib + 25 μM trametinib), and 25 μM 2155–14 and 2155–18 for 24 and 48 h. Manufacturer’s instructions for Muse™ PI3K/MAPK Dual Pathway Activation kit (EMD Millipore MCH200108) were followed. Briefly, cells were harvested, washed with PBS, and re-suspended in 1X assay buffer supplemented with fixation buffer and incubated on ice for 10 min. Cells were permeabilized with permeabilization buffer supplied with the kit and incubated with an antibody cocktail (5 μL of Anti-Akt/PKB + 5 μL of Anti-phospho-Akt). Cells were analyzed on Muse flow cytometer (EMD Millipore) using PI3K/MAPK Dual Pathway sub-routine.

### Annexin V flow cytometry assay

WM266–4 cells were seeded at 1, 000, 000 cells/flask in EMEM medium supplemented with 10% FBS and 1% penicillin/streptomycin in T25 flasks (Nunc cat# 75008384) and allowed to adhere overnight. 24 h after plating, the cells were pre-treated with pan-caspase inhibitor Z-VAD-FMK at 10 μM for 2 h. After pre-treatment, the cells were treated with 2155–14 (100 μM) and staurosporine (1 μM) for 4, 24, and 48 h. After 4, 24, and 48 h of compound exposure the adherent and floating cells were combined and stained using the TACS Annexin V-FITC Apoptosis detection kit (Trevigen, Inc., Gaithersburg, MD, USA) using the manufacturer’s protocol. Viable, necrotic, and early and late apoptotic cells were counted using an Accuri flow cytometer as per the manufacturer instructions.

### Cell staining for autophagy

WM266–4 cells were seeded at 10, 000 cells/well in 0.1 mL of E-MEM medium supplemented with 10% FBS and 1% penicillin/streptomycin in 96 well plates (Greiner Bio-One CellStar cat# 655180) and allowed to adhere overnight. After overnight incubation 100 μM 2155–14, 100 μM 2155–18, 50 nM hnRNP H2 siRNA, or 25 μM 2476–67.2 were added and incubated for various length of time. Cells were rinsed with warm PBS and stained with CYTO-ID® 1.0 Autophagy reagent (Enzo ENZ-51031–0050) and counterstained with DAPI. Cells were imaged using Cytation 5 imager (Biotek Inc, Winooski, VT) using GFP and DAPI filter sets. For % autophagic cell calculations cell counts were conducted using DAPI-stained nuclei and Object Sum Area value was obtained from at least 1, 000 cells/well using DAPI channels. To obtain the number of cells undergoing autophagy the cell count was conducted using the GFP channel. Both GFP and DAPI cell counts were conducted using an optimized algorithm in automatic mode. 8 replicate wells were used. % autophagic cells was calculated using Equation 1:

%autophagic cells=100%× (number of cells,GFP channel/number of cells,DAPI channel)

To calculate staining intensity, the Object Sum Area value from the GFP channel was obtained and divided by number of cells using the DAPI channel. Both GFP and DAPI cell counts were conducted using an optimized algorithm in automatic mode. 3 replicate wells were used.

### Mitochondrial potential assay

5, 000 WM266–4 cells were seeded in 100 μL of EMEM medium supplemented with 10% FBS in a 24-well plate. The cells were treated with carbonyl cyanide m-chlorophenyl hydrazone (CCCP, 25–50 μM) as a pharmacological control and 1–100 μM of 2155–14. After 1, 4, and 24 h, the cells were stained with 200 nM of Mito-Tracker dye (ThermoFisher cat# M22425) and DAPI for 1 h. Finally, the cells were washed with PBS and media was added. The cells were imaged and analyzed using a Cytation 5 imager using Cy5 and DAPI filter sets (Biotek Inc, Winoosky, VT). To calculate staining intensity, the Object Sum Area value from Cy5 channel was obtained and divided by the number of cells using the DAPI channel. Both Cy5 and DAPI cell counts were conducted using an optimized algorithm in automatic mode. 3 replicate wells were used.

### Western blotting for LC3

1 × 10^6^ of WM266–4 or M14 cells were seeded in 3 mL of E-MEM or RPMI-1640 medium supplemented with 10% FBS and 1% penicillin/streptomycin in 6 cm plates. After 24 h, the cells were pretreated with caspase inhibitors (2 μM Z-IETD-FMK, 2 μM Z-VEID-FMK, 10 μM Z-VAD-FMK) for 3 h. After pretreatment with caspase or autophagy inhibitors, the cells were treated with autophagy control (rapamycin (10 μM) and chloroquine (5 μM)) and apoptosis control (1 μM staurosporine) and 2155–14 and 2155–18 at various concentrations and length of time either with or without caspase or autophagy inhibitors. The cells were trypsinized (except for cells treated with fluorescent analogs of 2155–14, 2476–66.2 and 2476.67.2, which were scraped off the flasks) and collected in 15 mL tubes followed by lysis, SDS-PAGE, and western blot analysis of LC3 using polyclonal LC3 A/B antibody (Cell Signaling Cat# 4108, RP: 1:1000; 2% BSA) and actin using monoclonal β-actin antibody (Sigma-Aldrich A5441). After washing with TBST, the membranes were treated with chemiluminescent horseradish peroxidase detection reagent (Thermo Scientific, Cat# 32209) and exposed to autoradiography film (Denville Scientific, Inc., Metuchen, NJ, USA, cat# E3018). ImageJ software (NIH, Bethesda, MD) was used to quantify the intensity of proteins bands. The protein bands were normalized against loading controls (β-actin) and expressed as a fold of an untreated control.

### ER stress assay

0.2 × 10^6^ of WM266–4 cells were seeded in 3 mL of E-MEM supplemented with 10% FBS and 1% penicillin/streptomycin in 24-well plate. After 24 h, the cells were pre-treated either with 60 μM STF-83010 or vehicle for 3 h, after which cells were treated for 2 h with 5 μg/mL tunicamycin (ER stress inducer), 5 μM and 50 μM 2155–14. After 2 h, RNA was isolated using TRI Reagent® (Sigma #T9424) following the manufacturer’s protocol. Briefly, cells were washed with PBS and 0.5 mL of TRI reagent was added in each well and incubated at RT for 5 min. 0.1 mL of chloroform were added to each well and the plate was shaken for 9 sec and incubated at RT for 10 min. Next, the plate was centrifuged at 13, 000 rpm for 15 min at 4°C. Clear upper phase was transferred to fresh ultra-centrifuge tubes, 0.25 mL of isopropanol was added and mixed thoroughly, and the tubers were centrifuged at 13, 000 rpm for 10 min at 4°C. Supernatant was discarded and 0.5 mL of 75% ethanol was added to the tubes followed by vortexing and centrifugation at 13, 000 rpm for 5 min at 4°C. Supernatant was removed and the pellet was air-dried for 10 min. 30 μL of RNAse-free H_2_O were added and RNA concentration was measured by Nanodrop.

RT-PCR was conducted using Access RT-PCR system (Promega #A1260) following manufacturer’s protocol using forward (5’-cct tgt agt tga gaa cca gg-3’) and reverse (5’-ggg gct tgg tat ata tgt gg-3’) XBP1 primers (Biosynthesis Inc, Lewsville, TX). Amplified RNA was run in 2% agarose gel and stained with Gelgreen dye (Biotium #41004). Gels were imaged using LI-COR imager and quantified using Image Studio ™ Lite (LI-COR). The concentration of ER stress marker sXBP-1 was calculated as a percentage of total XBP-1 (total XBP-1 = sXBP-1 + XBP-1).

### Western blotting for Lamin A/C

1 × 10^6^ of WM266–4 or M14 melanoma cells were seeded in 3 mL of E-MEM or RPMI-1640 medium supplemented with 10% FBS and 1% penicillin/streptomycin in 6 cm plates. After 24 h, the cells were pre-treated with caspase inhibitors (2 μM Z-IETD-FMK, 2 μM Z-VEID-FMK, and 10 μM Z-VAD-FMK) for 3 h or autophagy inhibitors (10 μM LY294002 and 1 μM hydroxychloroquine) for 1 h. After pre-treatment with caspase or autophagy inhibitors, the cells were treated with apoptosis control (1 μM staurosporine) or autophagy inducer (10 μM rapamycin + 5 μM chloroquine) and 2155–14 at various concentrations and length of time either together or without caspase or autophagy inhibitors. The cells were trypsinized (except for cells treated with fluorescent analogs of 2155–14, 2476–66.2, and 2476.67.2, which were scraped off the flasks) and collected in 15 mL tubes followed by lysis, SDS-PAGE, and western blot analysis of Lamin A/C using polyclonal Lamin A/C antibody (Cell Signaling Cat# 2032, RP: 1:1000; 2% BSA) and actin using monoclonal β-actin antibody. After washing with TBST, the membranes were treated with chemilumiscent horseradish peroxidase detection reagent (Thermo Scientific, Cat# 32209) and exposed to autoradiography film (Denville Scientific, Inc., Metuchen, NJ, USA, cat# E3018). ImageJ software (NIH, Bethesda, MD) was used to quantify the intensity of proteins bands. The protein bands were normalized against loading controls.

### DARTS LC-MS/MS

WM266–4 melanoma cell lysates were prepared using a routine protocol with commercially available M-PER buffer supplemented with protease and phosphatase inhibitors. Lysates were split into control and compound test portions, and DMSO and 2155–14 were added to the respective lysate portions and incubated at RT for 1 h to allow for 2155–14 binding to its target(s). Pronase dilutions (1:100. 1:300, 1:1000. 1:3000 and 1:10000, Roche #10165921001) were prepared from 10 mg/mL stock. Lysates were split into 5 aliquots, and pronase dilutions were added to lysate aliquots and incubated at RT for 30 min. After 30 min, digestion was stopped by adding SDS loading buffer, and samples were heated up to 95 °C for 5 min and run on SDS-PAGE. After staining, one differentially hydrolyzed band was observed suggesting that binding of 2155–14 to the protein represented by the band made it less susceptible to the cleavage by pronase. Two gel bands were cut below the 75 kDa marker in the 1:1000 pronase dilution samples from the gels, in-gel treated with 10 mM DTT followed by 50 mM iodoacetamide, and subjected to trypsin digestion. Prior to mass spectrometry analysis, the peptide pools were acidified, desalted through Zip-Tip C18 tip columns and dried down. Each sample was reconstituted in 23 μL of 0.1% formic acid and 13 μL were utilized for MS analysis.

Each sample was analyzed by liquid-chromatography-tandem MS (LC-MS/MS) using an EASY-nLC 1000 system coupled to a Q Exactive mass spectrometer (Thermo Fisher Scientific). Peptides were concentrated and desalted on an RP pre-column (0.1 × 20 mm EASY-column, Thermo Fisher Scientific) and on-line eluted on an analytical RP column (0.075 × 100 mm EASY-column, Thermo Fisher Scientific), operating at 300 nL/min using the following gradient: starting at 5% B, 10% B for 3 min, 40–80% B in 60:00 min, 80% B in 6 min, and 5% B for 20 min [solvent A: 0.1% formic acid (v/v); solvent B: 0.1% formic acid (v/v), 80% ACN (v/v) (Fisher Scientific)]. Protein identification was carried out using Mascot algorithms, allowing optional modifications (Met oxidation), carbamidomethylation of Cys as a fixed modification, two missed cleavages, and a mass tolerance of 10 and 20 PPM for precursor and fragment ions, respectively. MS/MS raw files were searched against human proteins. Lamin A/C, DDX1, hnRNP H2, and hnRNP A2/B1 were additionally probed by western blot using methods described elsewhere in this section.

### Pull-down with biotinylated analogs of 2155–14

WM-266–4 and M14 melanoma cells were sonicated in RIPA lysis buffer containing added protease inhibitors. Affinity beads were prepared by addition of the 0.3 mg biotinylated probes to 50 μL of streptavidin agarose resin, which was then washed three times with the lysis buffer. Following incubation at 4 °C for 1 h, the obtained affinity beads complexed with biotinylated compounds were washed three times with lysis buffer to remove any unbound materials. The cell lysates (1 mg protein content) were then added to the probe-bound beads, and samples were incubated at 4 °C for 1 h and overnight on a rotator. Following 5 washes with lysis buffer, the samples were subjected to SDS-PAGE. The gels were stained overnight with colloidal blue (Invitrogen). Gel bands of interest were excised, reduced, carbidomethylated, dehydrated, and digested with Trypsin Gold (Promega) as per manufacturers’ instructions. Following digestion, peptides were extracted, all fractions were combined, the volume was reduced in a SpeedVac to near dryness, and peptides re-suspended to 20 μL using 95% ddH_2_O/5% ACN/0.1% formic acid (FA) prior to analysis by 1D reversed-phase LC-nESI-MS2 (as outlined below) [23].

### Mass spectrometry of pulldown bands

Peptide digests (8 μL each) were injected onto a 1260 Infinity nHPLC stack (Agilent) and separated using a 75 micron I.D. × 15 cm pulled tip C-18 column (Jupiter C-18 300 Å, 5 micron, Phenomenex). This system ran in-line with a Thermo Orbitrap Velos Pro hybrid mass spectrometer, equipped with a nanoelectrospray source (Thermo Fisher Scientific), and all data were collected in CID mode. The nHPLC was configured with binary mobile phases that included solvent A (0.1% formic acid in ddH_2_O) and solvent B (0.1% formic acid in 15% ddH_2_O/85% ACN) programmed as follows: 10 min @ 0%fet alB (2 μL/min, load), 90 min @ 0%−40%B (0.5 nL/min, analyze), 15 min @ 0%B (2 μL/min, equilibrate). Following each parent ion scan (350–1200 m/z @ 60k resolution), fragmentation data (MS2) was collected on the top most intense 15 ions. For data dependent scans, charge state screening and dynamic exclusion were enabled with a repeat count of 2, repeat duration of 30 s, and exclusion duration of 90 s.

### MS Data Conversion and Searches

The XCalibur RAW files were collected in profile mode, centroided, and converted to MzXML using ReAdW v. 3.5.1. The mgf files were then created using MzXML2Search (included in TPP v. 3.5) for all scans. The data was searched using SEQUEST, which was set for two maximum missed cleavages, a precursor mass window of 20 ppm, trypsin digestion, variable modification C at 57.0293, and M at 15.9949. Searches were performed with a species specific subset of the UniRef100 database.

### Peptide filtering, grouping, and quantification

The list of peptide IDs generated based on SEQUEST search results were filtered using Scaffold (Protein Sciences, Portland Oregon). Scaffold filters and groups all peptides to generate and retain only high confidence IDs while also generating normalized spectral counts (N-SC’s) across all samples for the purpose of relative quantification. The filter cut-off values were set with minimum peptide length of >5 AA’s, with no MH+1 charge states, with peptide probabilities of >80% C.I., and with the number of peptides per protein ≥2. The protein probabilities were then set to a >99.0% C.I., and an FDR<1.0. Scaffold incorporates the two most common methods for statistical validation of large proteome datasets, the false discovery rate (FDR) and protein probability [24–26]. Relative quantification across experiments was then performed *via* spectral counting [27, 28], and when relevant, spectral count abundances were then normalized between samples [29].

### Western blotting for hnRNP H2, DDX1, and hnRNP B1/A2

WM-266–4 cells were sonicated in in RIPA lysis buffer containing protease inhibitors. Affinity beads were prepared by addition of the biotinylated probes (0.3 mg) to the streptavidin agarose resin (50 μL), which was first washed three times with lysis buffer. Following incubation at 4 °C for 5 h, the obtained affinity beads were washed 3 times with lysis buffer to remove any unbound materials. The protein extract (1 mg) was then added to the probe-bound beads, and samples were incubated at 4 °C overnight. Following 5 washes with lysis buffer, the protein isolates were subjected to SDS-PAGE followed by transfer to nitrocellulose membrane. hnRNP H2 was detected using a rabbit polyclonal antibody (Abgent #: AP13497b; 1:3, 000, in 2% milk overnight), DDX1 using a monoclonal antibody (Santa Cruz # sc-271393, 1:1, 000 in 2% BSA overnight), and hnRNP B1/A2 using a monoclonal antibody (Santa Cruz # SC-32316, 1:1, 000 in 2% BSA overnight). After washing with TBST, the membranes were treated with chemilumiscent horseradish peroxidase detection reagent (Thermo Scientific, Cat# 32209) and exposed to autoradiography film (Denville Scientific, Inc., Metuchen, NJ, USA, cat# E3018). ImageJ software (NIH, Bethesda, MD) was used to quantify the intensity of proteins bands. The protein bands were normalized against loading controls.

### siRNA knockdown of hnRNP H2, DDX1, and hnRNP B1/A2

WM-266–4 cells (0.7 × 10^6^ cells in 500 μL) were seeded in a 60 mm dish. For each well to be transfected, RNAi duplex-Lipofectamine™ RNAiMAX complexes were prepared as follows. 10 μM RNAi duplex in 100 μL Opti-MEM® I was added in reduced Serum Medium without serum. The solution was mixed gently and incubated for 5 min. After incubation, 10 μL of Lipofectamine™ RNAiMAX was added to the mixture and mixed gently and then incubated for 30 min at RT. After incubation, RNAi duplex-Lipofectamine™ RNAiMAX complexes were added to each plate and incubated for 48 h at 37 °C, 95% relative humidity, 5% CO_2_. The medium was changed after 12–14 h. After 48 h, the cells were trypsinized and collected in 15 mL tubes followed by lysis and western blot analysis of hnRNP H2, hnRNP B1/A2, DDX1, and actin as shown in the previous section. Cell viability, LC3, and lamin A/C levels were assessed post knockdown using the above mentioned methods.

### Cell cycle arrest assay

3 × 10^6^ cells seeded in 5 mL of E-MEM medium supplemented with 10% FBS and 1% penicillin/streptomycin in 10 cm plates. After 24 h, the cells were harvested in 15 mL tubes, 2 × 10^6^ cells were fixed with 70% ice cold ethanol and stained using cell cycle reagent (Life Technologies # F10797). The cell cycle analysis was performed using Accuri flow cytometer (Biorad).

### Caspase 3/7, caspase 6, caspase 8, and caspase 9 activity assays

Briefly, WM266–4 and M14 cells were plated in 384-well plates in 5 μL of complete media (EMEM and RPMI-1640, respectively). 5 μL of 200 μM 2155–14 and 2155–18 were added to the cells. Plates were incubated at 37 °C, 5% CO_2_ and 95% relative humidity for various lengths of time. After incubation, 5 μL of caspase 3/7, caspase 8, and caspase 9 Glo® reagent (Promega cat#: G7570) were added to each well, and incubated for 15 min at room temperature. Luminescence was recorded using a Biotek Synergy H4 multimode microplate reader. In the case of caspase 6, 5 μL of 100 μM caspase 6 substrate AFC-VEID in lysis buffer was added and incubated for 1 h at 37 °C, 5% CO_2_ and 95% relative humidity and fluorescence intensity was measured at λ_excitation_ = 400 nm and λ_emission_ = 505 nm using a Biotek Synergy H4 multimode microplate reader.

### Calpain activity assays

The Calpain-Glo Protease Assay (Promega cat#) was used to measure the calpain enzyme activity in live cells, according to the manufacturer’s instructions. 1250 of WM-266–4 cells were plated in 384-well white TC-treated plates in 5 μL of serum-free EMEM media. The cells were incubated overnight at 37 °C, 5% CO_2_ and 95% relative humidity and 5 μL of test compounds 2155–14 (100 μM), calpain inhibitor III (25 μM), and PD151746 (25 μM) were added alone or in combination and incubated at 37 °C, 5% CO_2_, and 95% relative humidity for 0.5, 4, and 24 h. After incubation, 10 μL of a freshly prepared dilution of the Calpain-Glo Reagent stock solution (consisting of Suc-LLVY-Glo™ substrate, Calpain-Glo™ buffer, Luciferin Detection Reagent, and CaCl_2_) was added to cells at above mentioned times and luminescence was recorded with a Neo 2 microplate reader (Biotek Inc). All experiments were performed in triplicate and the values provided were the calculated average of at least three independent experiments.

## Results

### Melanoma cell panel profiling

Compounds 2155–14 and 2155–18 have been discovered as the result of the screen of three cell lines (M14 melanoma, A549 lung cancer, and CHO-K1 non-malignant control) [17]. Initial characterization demonstrated that these compounds caused late onset apoptosis. We tested additional melanoma cell lines harboring different mutations and non-malignant cells (Tables 1 and 2) to determine whether 2155–14 and 2155–18 preferentially inhibit melanoma cells with a specific molecular background which could provide further information about the mechanism of action. 2155–14 and 2155–18 did not exhibit significant preferences towards either of the tested melanoma cell lines (Table 2). 2155–14 and 215518 exhibited potency towards melanoma cell lines comparable to vemurafenib (marketed as Zelboraf) (Table 2 and [30, 31]). Also, similar to vemurafenib, there was limited toxicity towards non-malignant cells (Table 3). Activity across several melanoma cell lines with different mutational background and disease stages similar to vemurafenib suggested that 2155–14 and 2155–18 could potentially be used for broad-based melanoma therapy.

### Mechanism of cell death

**Fig. 1.**
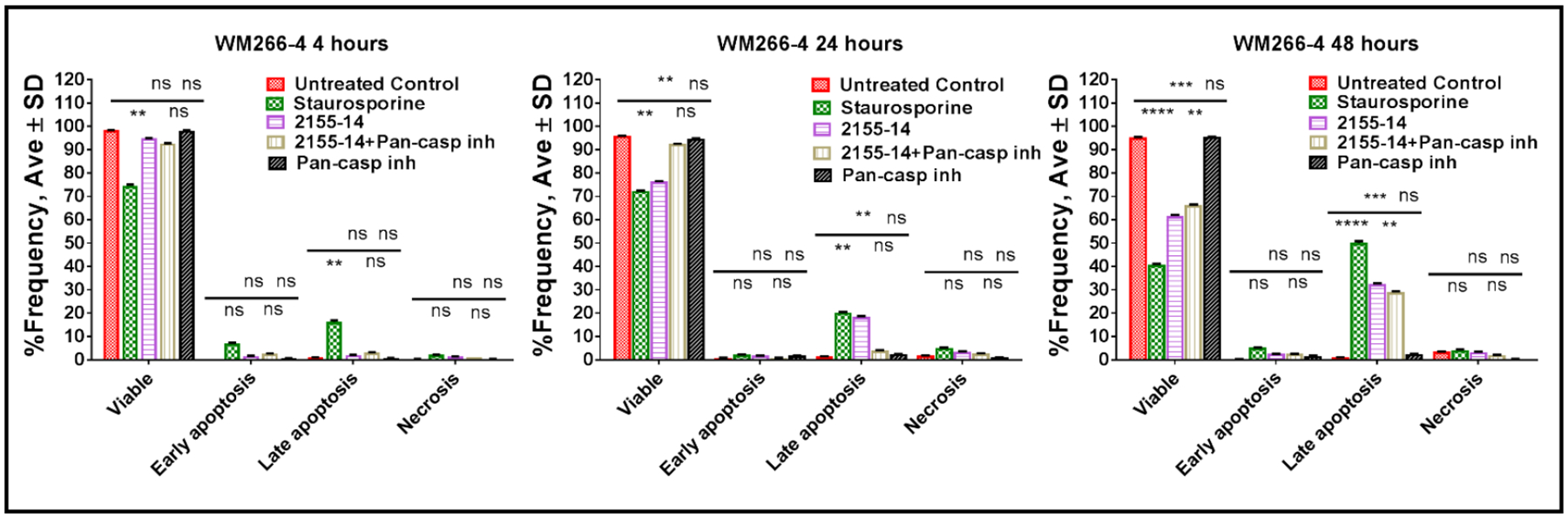
Annexin V assay confirms late apoptosis as one of the mechanisms of 2155–14-induced cell death. Starurosporine was used at 1 μM, 2155–14 was used at 100 μM, and pan-caspase inhibitor Z-VAD-FMK was used at 10 μM. Cells treated with 2155–14 and 2155–14+pan-caspase inhibitor have similar distribution of cell populations suggesting lack of pan-caspase inhibitor activity on biological effects of 2155–14 application.

Since 2155–14 and 2155–18 did not inhibit the viability of a variety of non-malignant cell types (Table 3), we further investigated the mechanism by which 2155–14 causes melanoma cell death. We previously reported caspase activity in response to 2155–14 treatment [17]. To ascertain if 2155–14-mediated increase of caspase activity leads to apoptosis we performed flow cytometry-based annexin V assays (Fig. 1). We chose WM266–4 cell line for more in-depth studies due its highly aggressive metastatic potential. Populations of viable, necrotic, early and late apoptotic WM266–4 melanoma cells were determined at 4, 24, and 48 h. At 4 h apoptosis was detected only in staurosporine-treated cells (Fig. 1, left panel). At 24 and 48 h, 18 and 32% of 2155–14-treated cells, respectively, underwent late apoptosis (Fig. 1, middle and right panels), consistent with our prior hypothesis [17]. Early apoptotic and necrotic cells were less than 5% of the total population, suggesting that 2155–14 causes late apoptosis. At 24 h the combination of 2155–14 and pan-caspase inhibitor had lower amounts of cells undergoing late apoptosis as compared to 2155–14 alone (4 and 20%, respectively). However, at 48 h, the combination of 2155–14 with pan-caspase inhibitor and 2155–14 alone showed similar levels of cells in late apoptosis (27 and 29%, respectively). This suggests that even though cell death occurs *via* apoptosis caspase inhibition can only slow it down but not stop it completely.

**Fig. 2.**
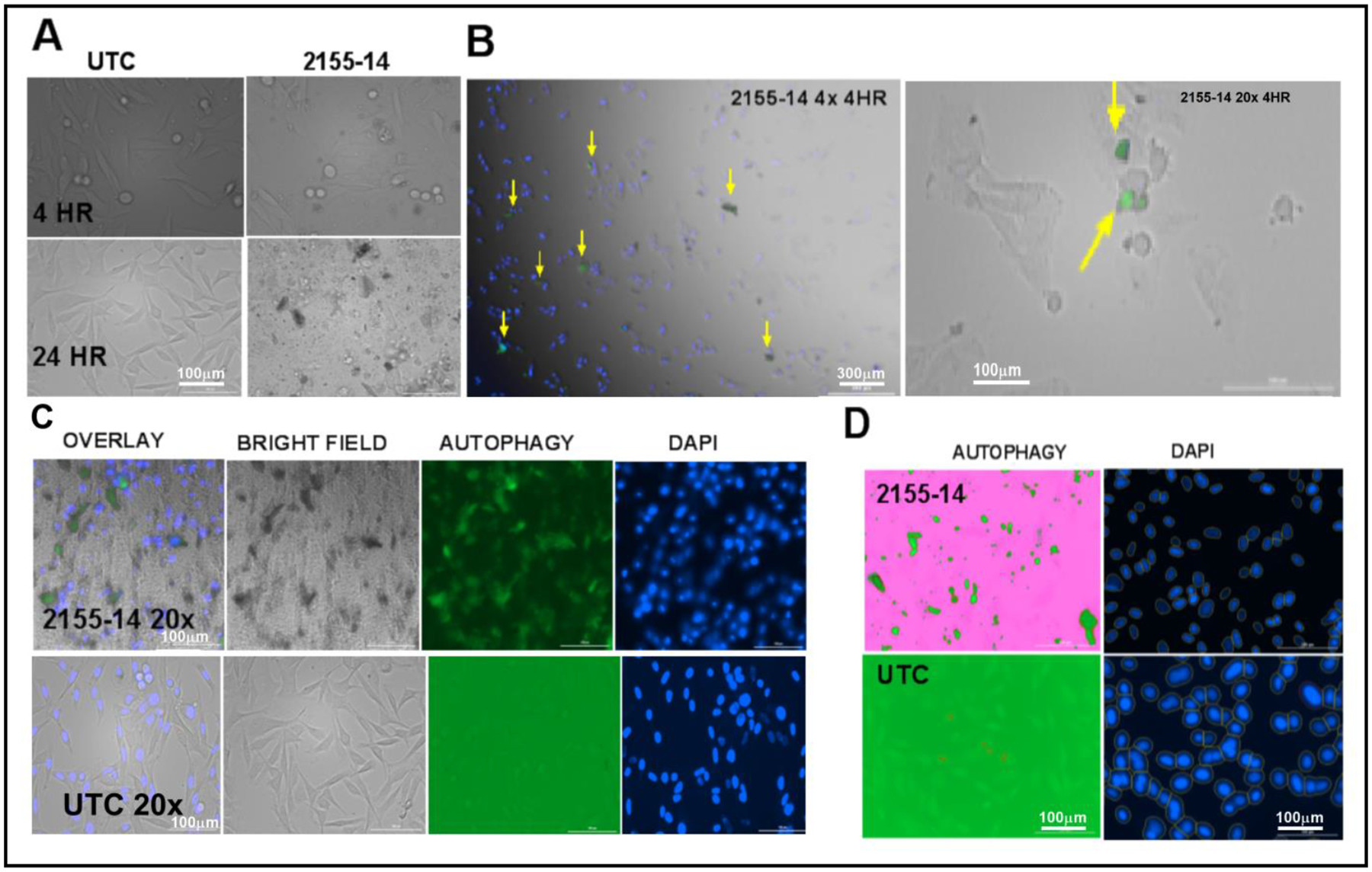
Autophagy detection in WM266–4 cells using autophagosome dye. (A) Bright field micrographs at 4x magnification of unstained WM266–4 cells in the presence of 2155–14 and 2155–18 4 and 24 h after compound addition. Please note differences in cell morphology as compared to untreated control (UTC). (B) WM266–4 cells stain positive for autophagy at 4 h after addition of 2155–14, but not 2155–18. Nuclei are stained blue. Yellow arrows indicate green puncta signifying autophagosome formation. (C) WM266-4 cells show increased autophagy staining at 24 h after addition of 2155–14, but not 2155–18. Nuclei are stained blue. (D) Autophagy (GFP) channel was used to quantify positive WM266–4 cells. Number of cells present in each well was normalized using DAPI-stained nuclei. Scale bar = 100 μm for all images with 20x magnification and 300 μm for all images with 4x magnification.

To further investigate the mechanism of cell death, we stained WM-266–4 cells treated with 2155–14 for autophagy and mitochondrial potential at 1, 4, and 24 h after addition of compound. At the 1 h time point we did not observe either autophagy or other signs of cellular distress (data not shown). At the 4 h time point unstained cells treated with 2155–14 exhibited morphologic traits of apoptosis (Fig. 2A, top panel; note smaller cell size, rounded shape, membrane blebbing, and apoptotic bodies). Also, at 4 h cells stained positive for autophagosomes (Fig. 2B, left panel). At 24 h the apoptosis intensified leading to extensive cell death in the case of 2155–14 treatment (Fig. 2A, lower right panel; Fig. 2B, right panel). At 24 h the intensity of autophagy staining increased in cells treated with 2155–14 (Fig. 2C). Quantitation of the representative image showed that 57% of cells treated with 2155–14 were undergoing autophagy, whereas untreated control cells showed no signs of autophagy (Fig. 2D).

**Fig. 3.**
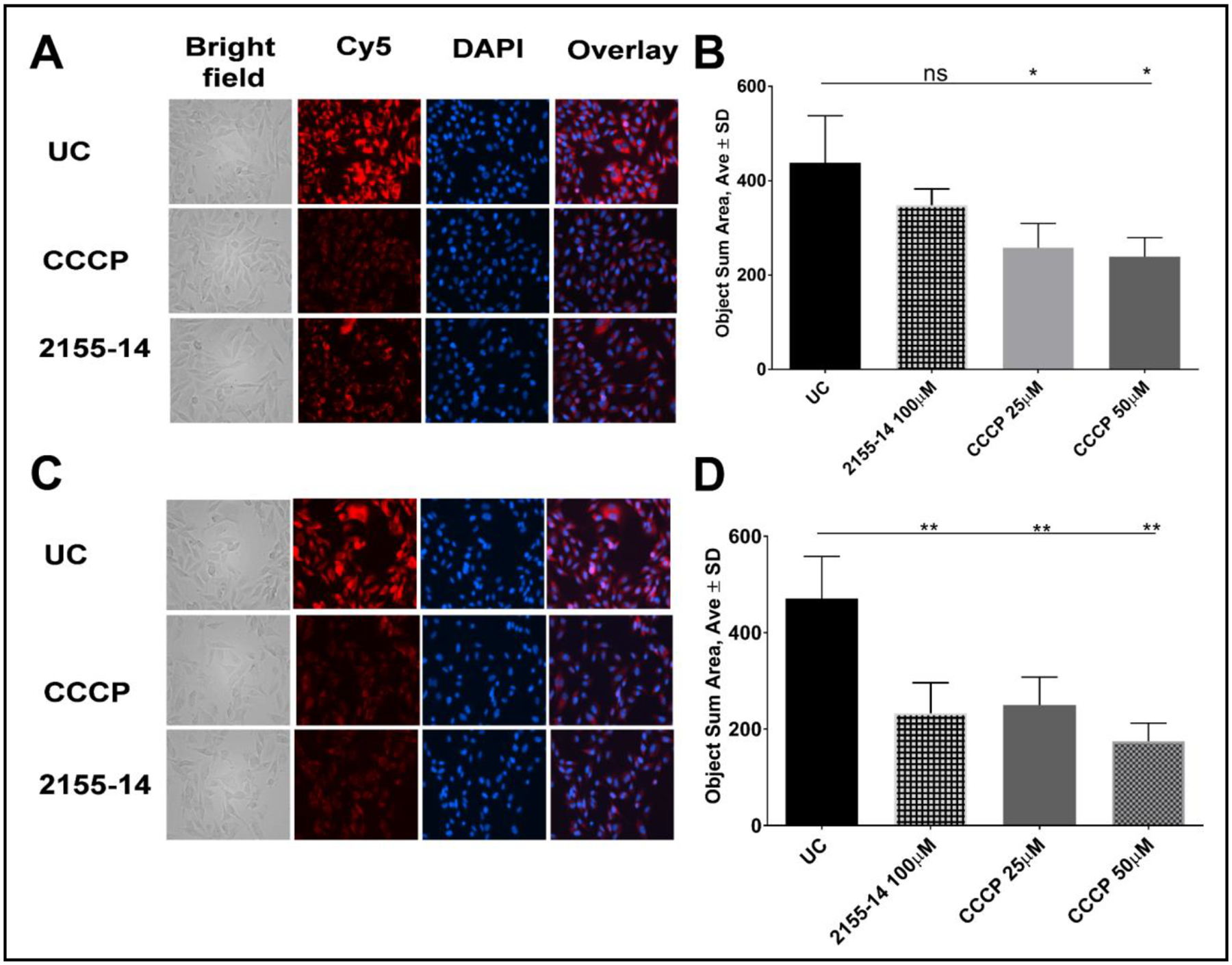
Effect of 2155–14 on mitochondrial potential of WM266–4 cells. (A). Representative images of cells 1 h after addition of compounds; (B) Quantitation of effects of compounds on mitochondrial potential 1 h after compound addition. There is no significance between untreated control and cells treated with 2155–14. One-way analysis of variance (ANOVA) was used followed by Dunnett post hoc test. The data shown are the mean ± SD, n = 4. * - p<0.05; (C). Representative images of cells 4 h after addition of compounds; (D) Quantitation of effects of compounds on mitochondrial potential 1 h after compound addition. One-way analysis of variance (ANOVA) was used followed by Dunnett post hoc test. The data shown are the mean ± SD, n=4. ** - p<0.01; UC – untreated control, CCCP - carbonyl cyanide m-chlorophenyl hydrazine.

Staining for mitochondrial potential (Fig. 3) was performed using carbonyl cyanide m-chlorophenyl hydrazine (CCCP) as a positive control, as CCCP is an inhibitor of oxidative phosphorylation. At 1 h, mitochondrial potential was only slightly decreased in the presence of 2155–14, and the difference was not significant (Fig. 3A and B). At 4 h, mitochondrial potential was significantly decreased in the presence of 2155–14 (Fig. 3C and D).

**Fig. 4.**
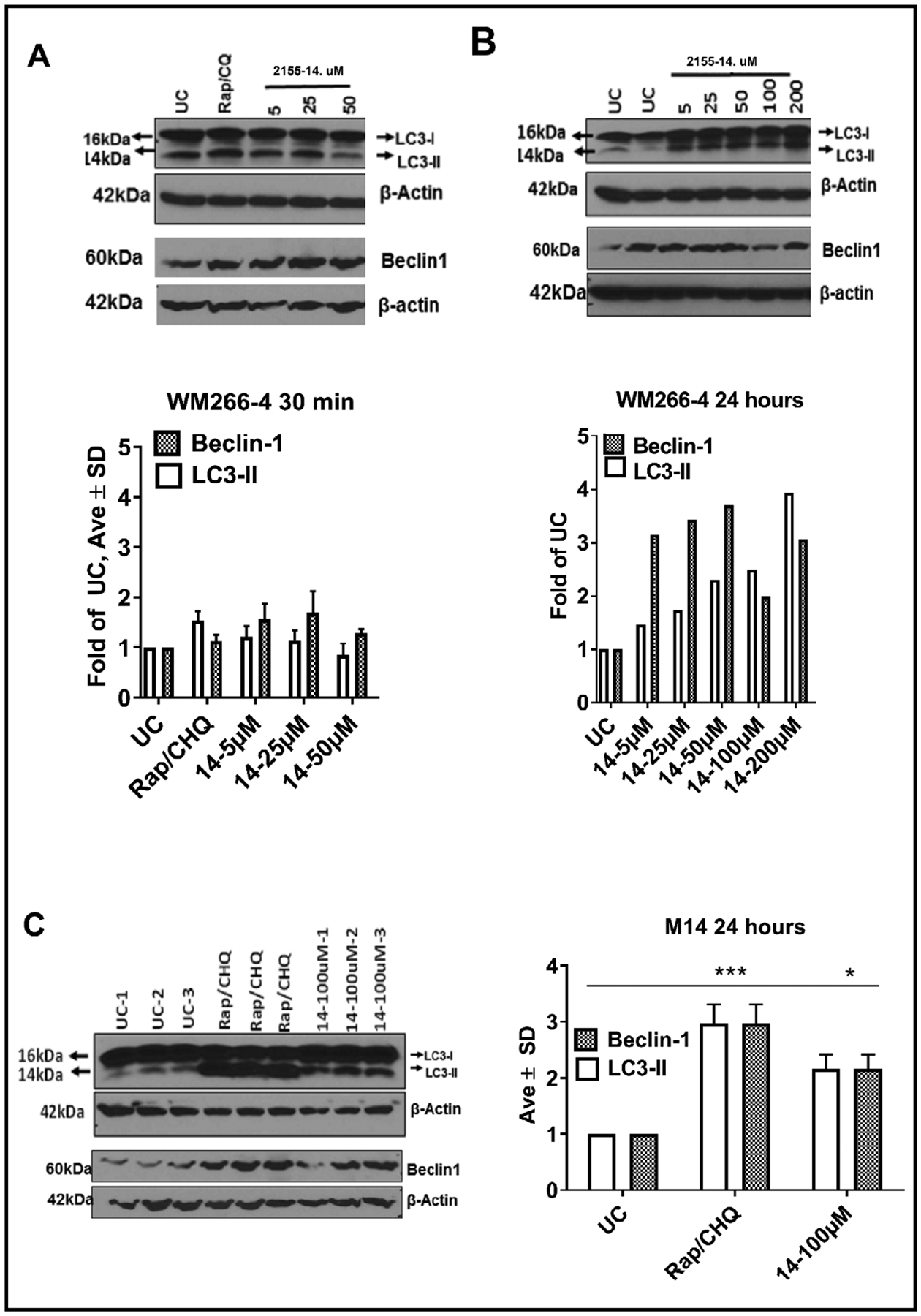
Western blot analysis of WM266–4 and M14 melanoma cells confirms that autophagy is induced by 2155–14. (A) LC3 and beclin-1 representative blots and quantification 30 min after addition of 5–50 μM 2155–14 and 2155–18 to WM266–4 cells. No statistical significance between untreated control and test conditions was observed. (B) LC3 and beclin-1 representative blots and quantification 24 h after addition of 5–200 μM 2155–14 to WM266–4 cells. Dose dependent increase of LC3 and beclin-1 was observed in the case of 2155–14, but not 2155–18. (C) LC3 and beclin-1 representative blots and quantification 24 h after addition of 100 μM 2155–14 to M14 cells. UC – untreated control. Rapamycin/chloroquine (Rap/CHQ) mixture used as a control. One-way analysis of variance (ANOVA) was used followed by Dunnett post hoc test. The data shown were the mean ± SD, n=3. ***** - p<0.0001, *** - p<0.001, ** - p<0.01, * - p<0.05.

We further investigated 2155–14-induced autophagy by western blotting of autophagy markers LC3-II [32] and beclin-1 in both WM266–4 and M14 melanoma cells (Fig. 4). Since we observed autophagy staining at 4 h after treatment with 2155–14 (Fig. 2) we examined how soon after compound treatment the levels of LC3-II increased. At an early time point (30 min) we did not observe autophagy potentiation as compared to the untreated control (Fig. 4A). 24 h after 2155–14 treatment we observed a dose dependent increase of LC3-II, beclin-1, and autophagy in WM266–4 cells (Fig. 4B and 3E) and an increase in LC3-II and beclin-1 in M14 cells (Fig. 4C).

**Fig. 5.**
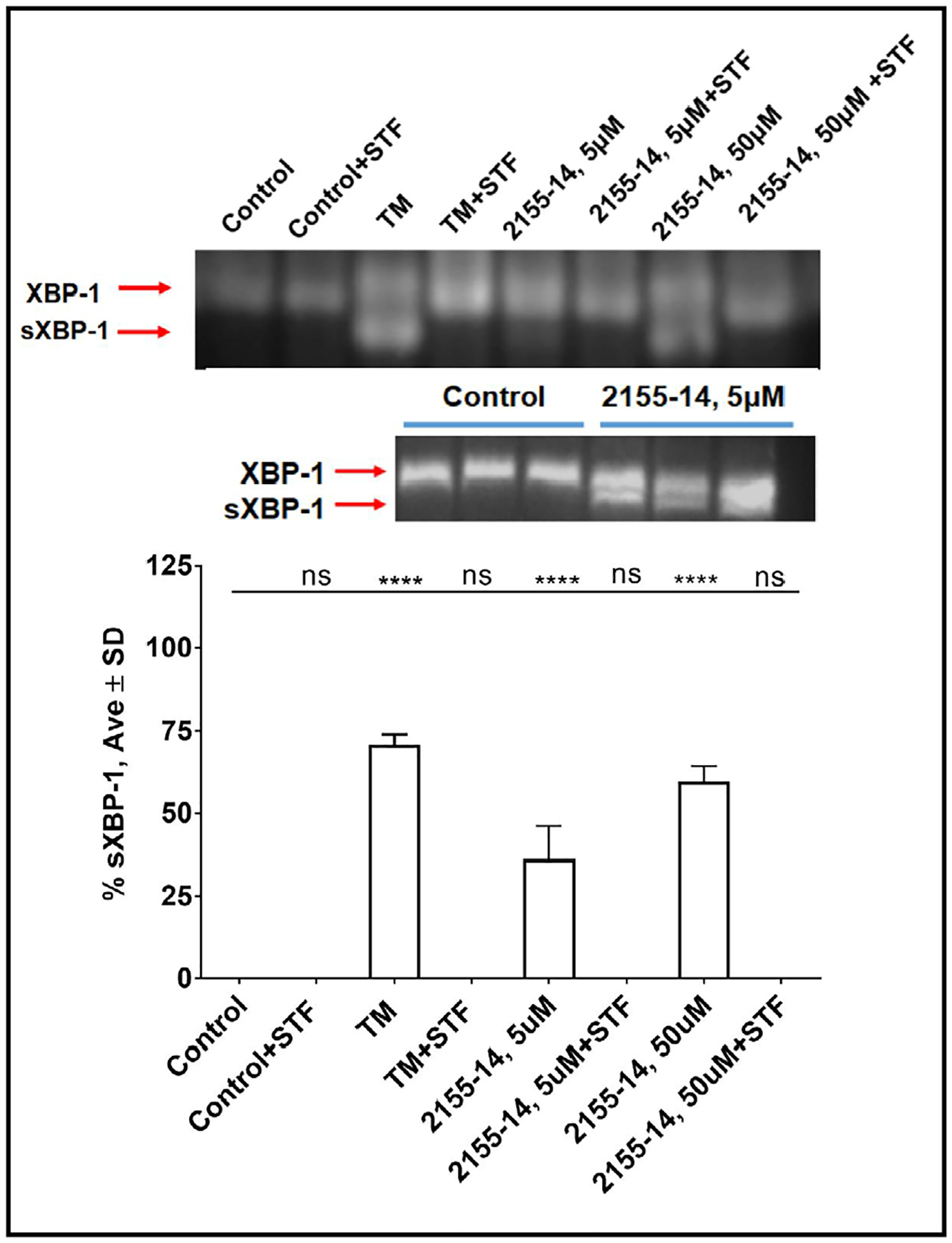
RNA blot analysis of ER stress marker sXBP-1 in WM266–4 cells confirms that 2155–14 induces ER stress. Representative blot and quantification 2 h after addition of 5 μM and 50 μM 2155–14 to WM266–4 cells. % sXBP-1 was calculated as a % of total XBP-1 (total XBP-1 = sXBP-1 (lower band) + XBP-1 (upper band)). sXBP-1 band in 5 μM 2155–14 lane was confirmed by running additional replicates (lower blot). 5 μg/mL tunicamycin (TM) used as a positive control. STF – 60 μM STF-83010, an inhibitor of IRE1 RNase activity responsible for splicing of XBP1 into ER stress marker sXBP1. One-way analysis of variance (ANOVA) was used followed by Dunnett post hoc test. The data shown were the mean ± SD, n=3. **** - p<0.0001, ns – no significance.

ER stress has been shown to initiate both autophagy and changes in mitochondrial potential leading to apoptosis [33]. Therefore, we tested for a marker of ER stress, sXBP1 [34], 2 h after addition of 2155–14. We used 5 μM (~IC_50_ for WM266–4) and 50 μM 2155–14. Both 5 and 50 μM 2155–14 induced sXBP1 formation (sXBP1 constituted 36 ± 10 and 60 ± 5% of total XBP1, respectively) (Fig. 5). sXBP1 formation by 2155–14 was inhibited by 60 μM STF-83010 (Fig. 5), where STF-83010 is an inhibitor of IRE1 RNase activity responsible for splicing of XBP1 into ER stress marker sXBP1 [35].

### Pathway analysis

**Fig. 6.**
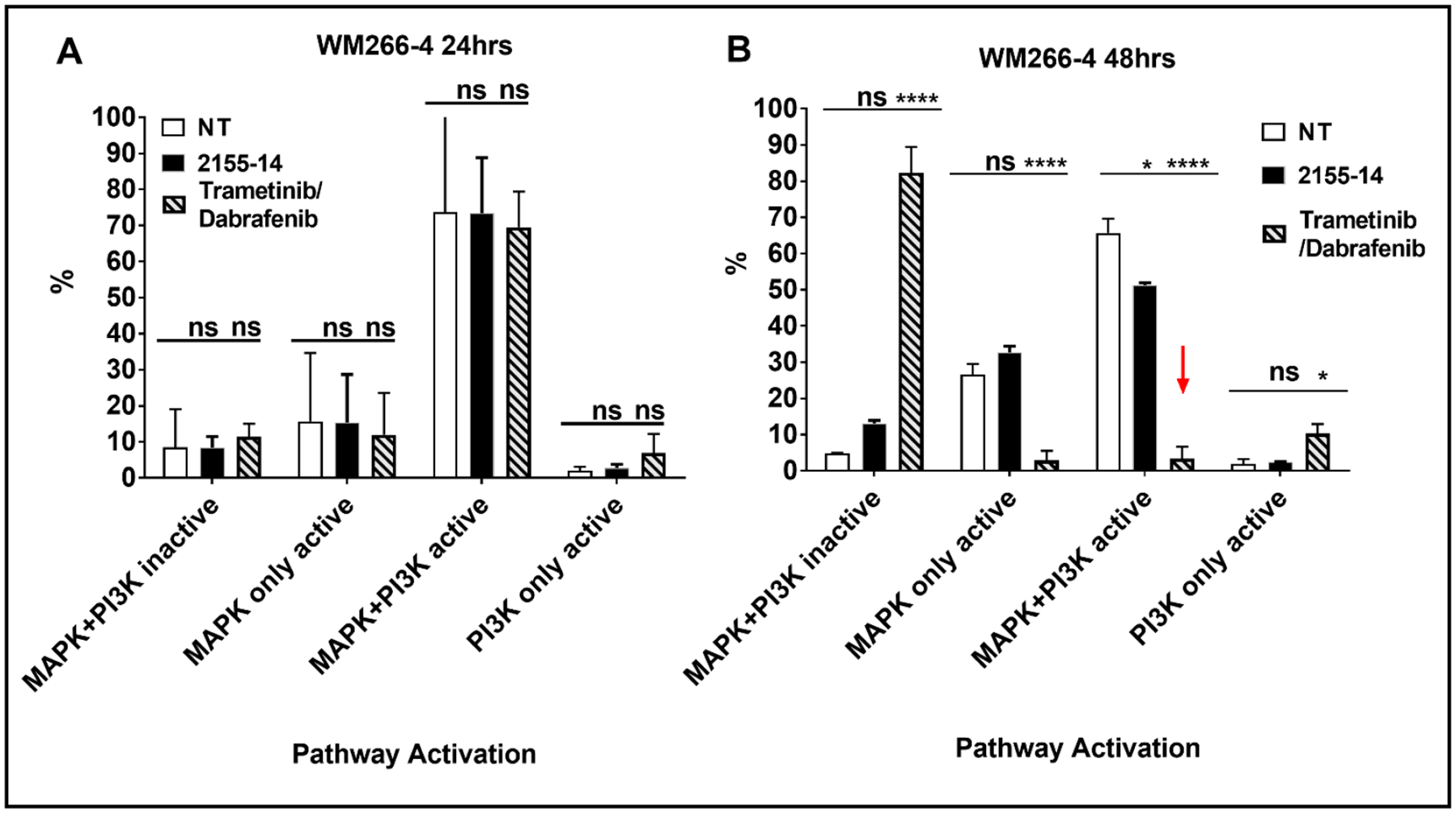
Results of flow cytometry-based assay of MAPK and PI3K pathway activation of WM266–4 cells in presence of 2155–14. (A). 24 h treatment with 100 μM 2155–14. (B) 48 h treatment with 100 μM 2155–14. One-way analysis of variance (ANOVA) was used followed by Dunnett post hoc test. The data shown were the mean ± SD, n=3. ***** - p<0.0001, ns – no significance; 2155–14 Trametinib/dabrafenib combination was tested at 25 μM of each compound. MAPK+PI3K inactive – cell population with neither MAPK nor PI3K pathway activated; MAPK only active - cell population with just MAPK pathway activated; MAPK+PI3K active - cell population with both MAPK and PI3K pathways activated; PI3K only active - cell population with just PI3K pathway activated.

We utilized flow cytometry in order to determine if 2155–14 had an effect on the Ras/Raf/MEK/ERK pathway (Fig. 6). At the 24 h 2155–14 did not exhibit any effect on phosphorylation of ERK 1/2 or Akt (Fig. 6A). At 48 h, cell treatment with 2155–14 resulted in approximately a 20% decrease of cell population with dual pathway activation as compared to the untreated control (Fig. 6B). Cells with single pathway activation were not significantly affected (Fig. 6B) while the number of cells where both pathways were inactive increased by approximately 10%. Treatment with the combination of BRAF and MEK1/MEK2 inhibitors dabrafenib/trametinib strikingly decreased the population with dual pathway activation which inversely correlated with population with dual inactive pathways, but unlike 2155–14 the amount of PI3K-activated cells was increased (Fig. 6B). There is a significant cross-talk between PI3K and MAPK pathways, and therefore the increased amount of PI3K-activated cells in response to MEK inhibition is not surprising and has been reported by others as a basis of acquired resistance to BRAFi/MEKi therapy [11–13, 36]. Therefore, the absence of PI3K activation in cells treated with 2155–14 could be beneficial for melanoma therapy.

### Target ID studies

**Fig. 7.**
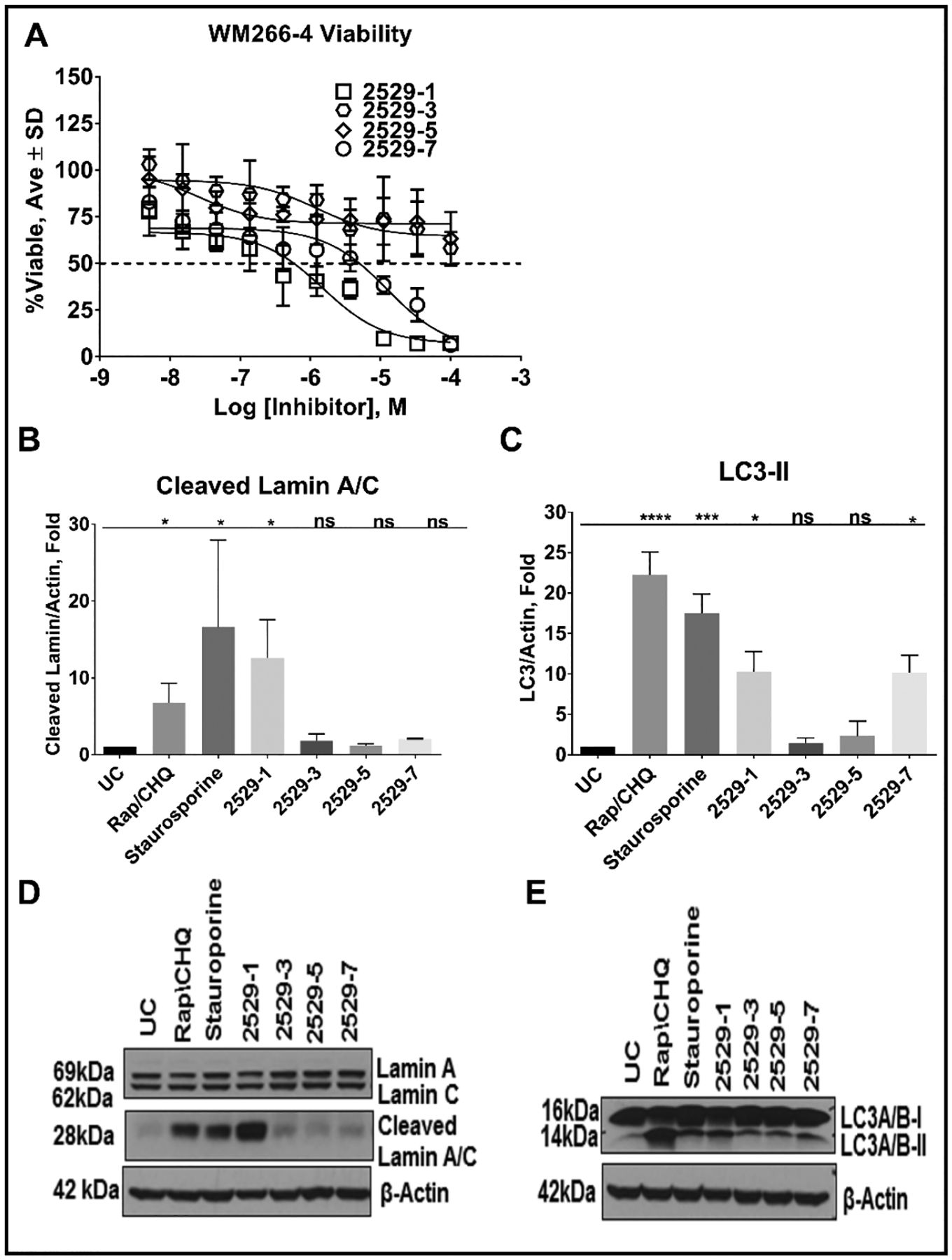
Effect of 2155–14 and its biotinylated analog on cell viability, levels of LC3 and cleaved lamin A/C in WM266–4 cells. (A) Results of cell viability study after 72 h treatment. (B) Quantification of western blot of cleaved lamin A/C in presence of 100 μM 2155–14/2529–1, 2529–3, 2529–5, and 2529–7. (C) Quantification of western blot of LC3-II in presence of 100 μM 2155–14/2529–1, 2529–3, 2529–5, and 2529–7. (D) Representative western blot of lamin A/C in presence of 100 μM 2155–14/2529–1, 2529–3, 2529–5, and 2529–7. (E) Representative western blot of LC3 in presence of 100 μM 2155–14/2529–1, 2529–3, 2529–5, and 2529–7. One-way analysis of variance (ANOVA) was used followed by Dunnett post hoc test. The data shown are the mean ± SD, n=3. ***** - p<0.0001, *** - p<0.001, ** - p<0.01, * - p<0.05, ns - not significant. Rap/CHQ = Rapamycin (5 μM)/Chloroquine (10 μM). Staurosporine was used at 1 μM. Note that 2529–3 and 2529–5 did not significantly increase levels of cleaved lamin A/C and LC3-II explaining their lower potency against WM266–4 cells. 2529–7 increased LC3-II to the levels of 2155–14/2529–1, while failing to increase levels of cleaved lamin A/C.

Pathway analysis did not reveal a candidate molecular target for 2155–14. To identify target(s) of 2155–14, we re-synthesized compound 2155–14 (Supplementary Table S1, 2529–1) and analogs of 2155–14 (Supplementary Table S1, 2529–3, 2529–5, and 2529–7) biotinylated in positions 1–3 to enable pulldown studies. We compared the effects of 2155–14 and biotinylated analogs on melanoma cell viability and levels of LC3-II and lamin A/C cleavage to ascertain if biotinylation resulted in significant changes of biological activities. 2529–1 (resynthesis of 2155–14) and 2529–7 (analog of 2529–1 biotinylated in position 3, Supplementary Table S1) were as potent as 2155–14 in inhibiting the viability of WM266–4 cells (Fig. 7A, IC_50_ = 1.5 ± 0.2, 4 ± 0.5, and 3.3 ± 0.5 μM for 2529–1, 2155–14, and 2529–7, respectively). 100 μM of either 25291 or 2529–7 completely inhibited the viability of WM266–4 cells after 72 h of treatment (Fig. 7A). For comparison, 2529–3 and 2529–5 were significantly less potent than 2529–1 and 2529–7 (IC_50_ > 100 μM), exhibiting maximal inhibition of cell viability of only 42% and 37% at 100 μM at 72 h, respectively (Fig. 7A). Western blot analysis revealed that biotinylated compounds 2529–3, 25295, and 2529–7 had no effect on cleavage of apoptosis marker lamin A/C as opposed to non-biotinylated 2155–14/25291 (Fig. 7B and D). Interestingly, only biotinylated 2529–7 demonstrated an effect on LC3-II levels similar to non-biotinylated 2155–14/2529–1 (Fig. 7C and E). This suggested that 2529–7 induces autophagy but not apoptosis at 24 h after exposure, potentially binding targets relevant to induction of autophagy but not apoptosis. Additionally, this explains the significantly lower effect of 2529–3 and 2529–5 on inhibition of WM266–4 cell viability.

**Fig. 8.**
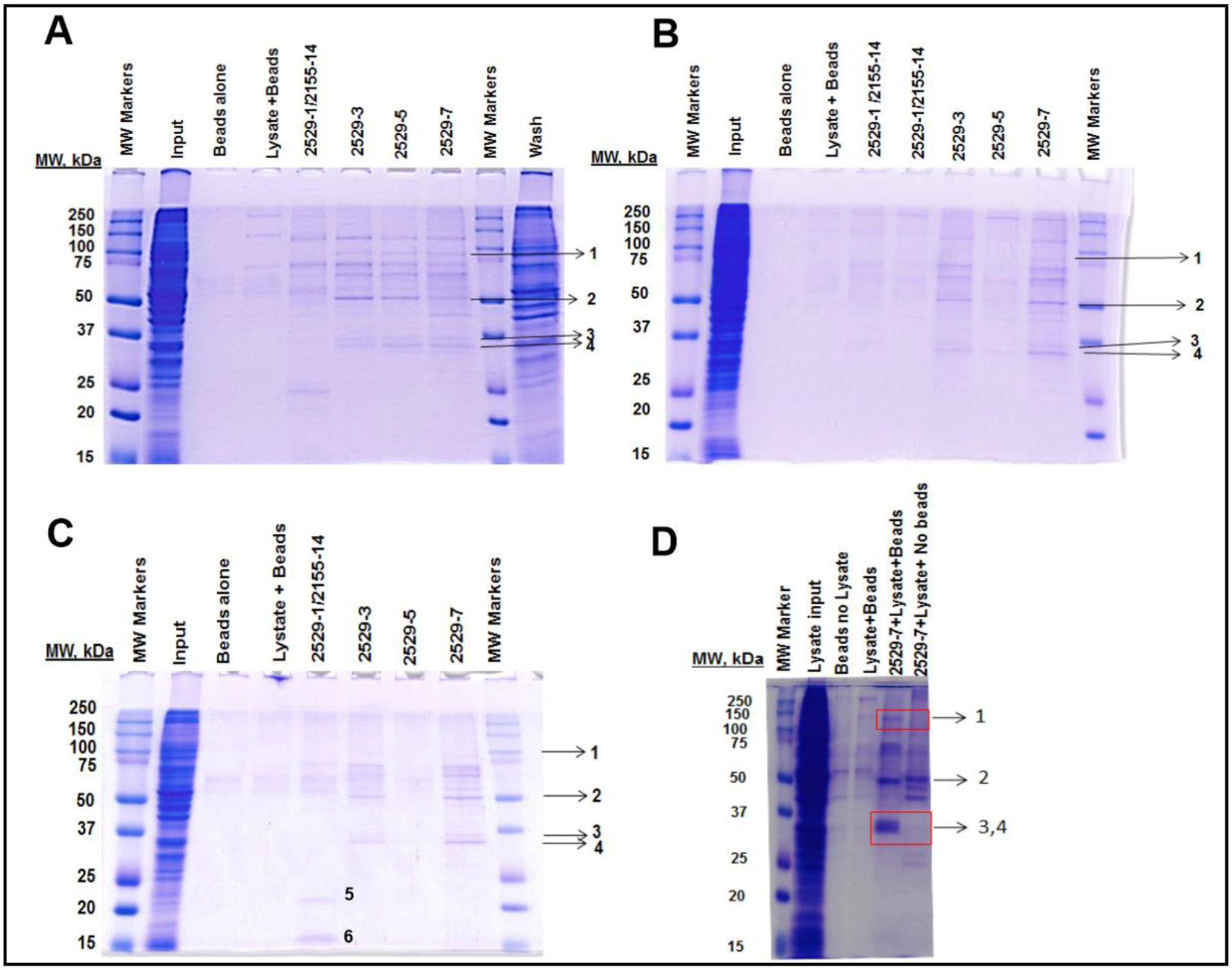
SDS-PAGE of pulldown of WM266–4 cell lysates incubated with biotinylated analogs of 2155–14 pre-complexed with streptavidin beads. (A) WM266–4 cell lysates incubated for 24 h. (B) M14 cell lysates incubated for 24 h. (C) WM266–4 cell lysates incubated for 1 h. Note that 1 h incubation is missing band 1 present in 24 h incubation experiments in either WM266–4 or M14 cells. Note that bands 1–4 appear in both tested melanoma cell lines suggesting a common target for 2155–14. (D) Repeat pulldown experiment with 2529–7 confirms bands 1–4, but not bands 5–6. Bands 1–4 were excised and sent for identification. Pulldown experiment was repeated three times. Protein identification was performed twice on bands from two independent pulldown experiments.

We conducted pulldown of whole cell lysates of WM266–4 and M14 melanoma cells using biotinylated analogs of 2155–14 complexed to streptavidin agarose beads. Lysates were incubated with beads complexed to biotinylated analogs of 2155–14 for 1 and 24 h. We compared protein bands found in lanes representing beads complexed to biotinylated analogs of 2155–14 (2529–3, 2529–5, and 2529–7) to control lanes (beads alone, lysate + beads, and non-biotinylated 2155–14/2529–1). Bands occurring in both control and sample lanes were not pursued any further. As evidenced by SDS-PAGE gels (Fig. 8A and B), four specific bands (occurring only in 2529–3, 2529–5 and/or 2529–7 treatment lanes) were found in lysates of WM266–4 and M14 melanoma cells following 24 h incubation with the respective compound-beads. Interestingly, bands 2–4 were also found in WM266–4 lysate incubated for 1 h with beads complexed to biotinylated analogs of 2155–14, while band 1 was absent suggesting slower binding. In addition to four specific bands found only in lanes for 2529–3, 2529–5, and 2529–7, treatment with non-biotinylated 2155–14/2529–1 resulted in two bands that were not found in any other lane (Fig. 8C, bands 5 and 6). However, bands 5 and 6 did not appear in the repeat of this experiment and were not further pursued (data not shown).

We repeated the pulldown experiment for 24 h incubation of lysate and beads complexed to 2529–7. The same 4 specific bands were found (Fig. 8D), and thus we proceeded with protein identification by proteomics.

Proteomic analysis of bands 1–4 revealed top hits of ATP-dependent RNA helicase DDX1 (band 1, accession #Q92499), heterogeneous nuclear ribonucleoprotein H2 (band 2, hnRNP H2, accession #P55795), and heterogeneous nuclear ribonucleoprotein A2/B1 (bands 3 and 4, hnRNP A2/B1, accession # P22626) (Table 4). hnRNP A2 is a shorter isoform of canonical hnRNP B1, lacking residues 3–14 as compared to the canonical sequence. The lack of coverage in the 3–14 amino acid region in band 4 as compared to band 3 further corroborates this hypothesis (Supplementary Fig. 2). This data suggested that band 3 (Fig. 8A) is hnRNP B1, while band 4 is likely hnRNP A2.

**Fig. 9.**
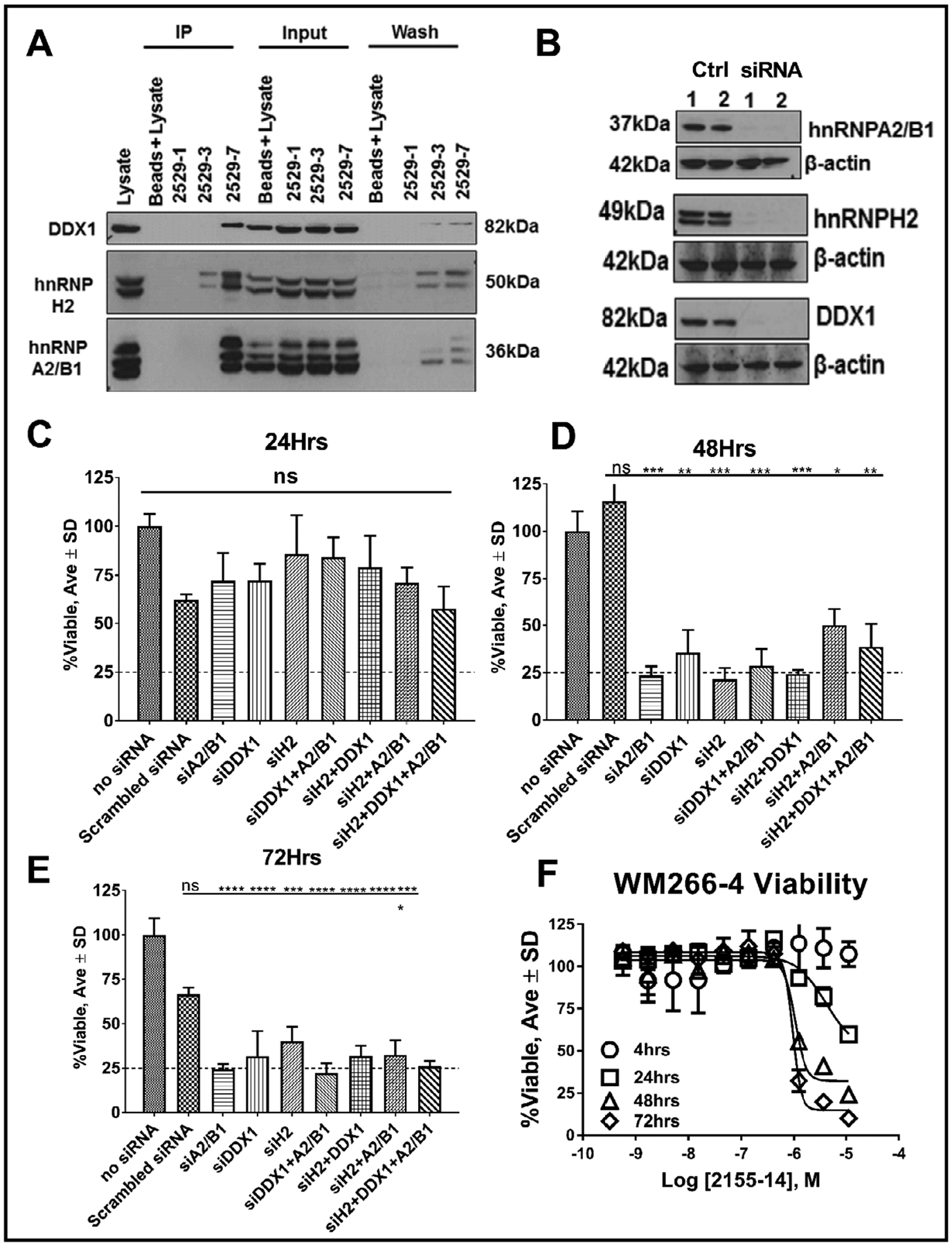
Genomic confirmation of DDX1, hnRNP H2, and hnRNP A2/B1 as targets of 2155–14. (A) Western blot of WM266–4 lysates validates DDX1, hnRNP H2, and hnRNP A2/B1 as binding targets of biotinylated analog of 2155–14, 2529–7. (B) DDX1, hnRNP H2, and hnRNP A2/B1 expression is knocked down by the corresponding siRNAs. (C,D,E) WM266–4 cell viability after treatment with DDX1, hnRNP H2, and hnRNP A2/B1 siRNAs and combinations thereof at concentrations which produced complete knockdown of respective proteins as shown in (B). One-way analysis of variance (ANOVA) was used followed by Dunnett post hoc test. The data shown were the mean ± SD, n=6. ***** - p<0.0001, *** - p<0.001, ** - p<0.01, * - p<0.05, ns = no significance. (F) WM266–4 viability time course assay in the presence of dose response of 2155–14. Please note similarity of time dependent loss of viability of WM266–4 starting at 48 h in the presence of siRNAs and 2155–14.Please note similarity of time dependent loss of viability of WM266–4 starting at 48 h in the presence of siRNAs and 2155–14.

Western blot analysis of the pulldown SDS-PAGE gel was next performed. We were able to confirm the identity of DDX1 using a monoclonal antibody (Fig. 9A, top strip, predicted MW of 82 kDa) in the case of 2529–7 treatment, but not 2529–3 treatment. To our surprise, in the case of hnRNP H2, we detected two bands in both 2529–3 and 2529–7 treatment lanes (Fig. 9A, middle strip). Due to the lack of commercially available monoclonal antibodies for hnRNP H2, we utilized a polyclonal antibody raised to the region 311–340 of hnRNP H2. This region is found in several proteins from hnRNP family, including hnRNP H1, which was the second highest scored protein after hnRNP H2 identified in band 2 of the proteomics experiment (Table 4). ClustalW alignment showed that hnRNP H1 and H2 shared 96% amino acid identity based on the total sequence. Additionally, 29 out 30 amino acids in the antigen region (311–340) were identical indicating that the proteomics approach was not capable of resolving these proteins and 2155–14 could be interacting with both proteins (for the purposes of this report we will be referring to hnRNPH2). Interestingly, hnRNPH2 bands in the 2529–3 lane were much less abundant despite similar intensity in the input lanes suggesting that interactions between hnRNP H2 and 2529–3 are weaker than between hnRNP H2 and 2529–7. This could be the reason for the lower activity of 2529–3 against melanoma cells compared with 2529–7. Application of hnRNP A1/B2 monoclonal antibody revealed three bands in the 2529–7 treatment lane, but no bands were found in the 2529–3 treatment lane, suggesting a selective binding of 2529–7 to hnRNP A2/B1 (Fig. 9A, bottom strip). As mentioned above, band 3 in the SDS-PAGE can be attributable to hnRNP B1, while band 4 is likely a hnRNP A2 isoform. An appearance of the third band is, therefore, unexpected and could be suggestive of binding of 2529–7 to a protein which was not detected by LC-MS/MS.

**Fig. 10.**
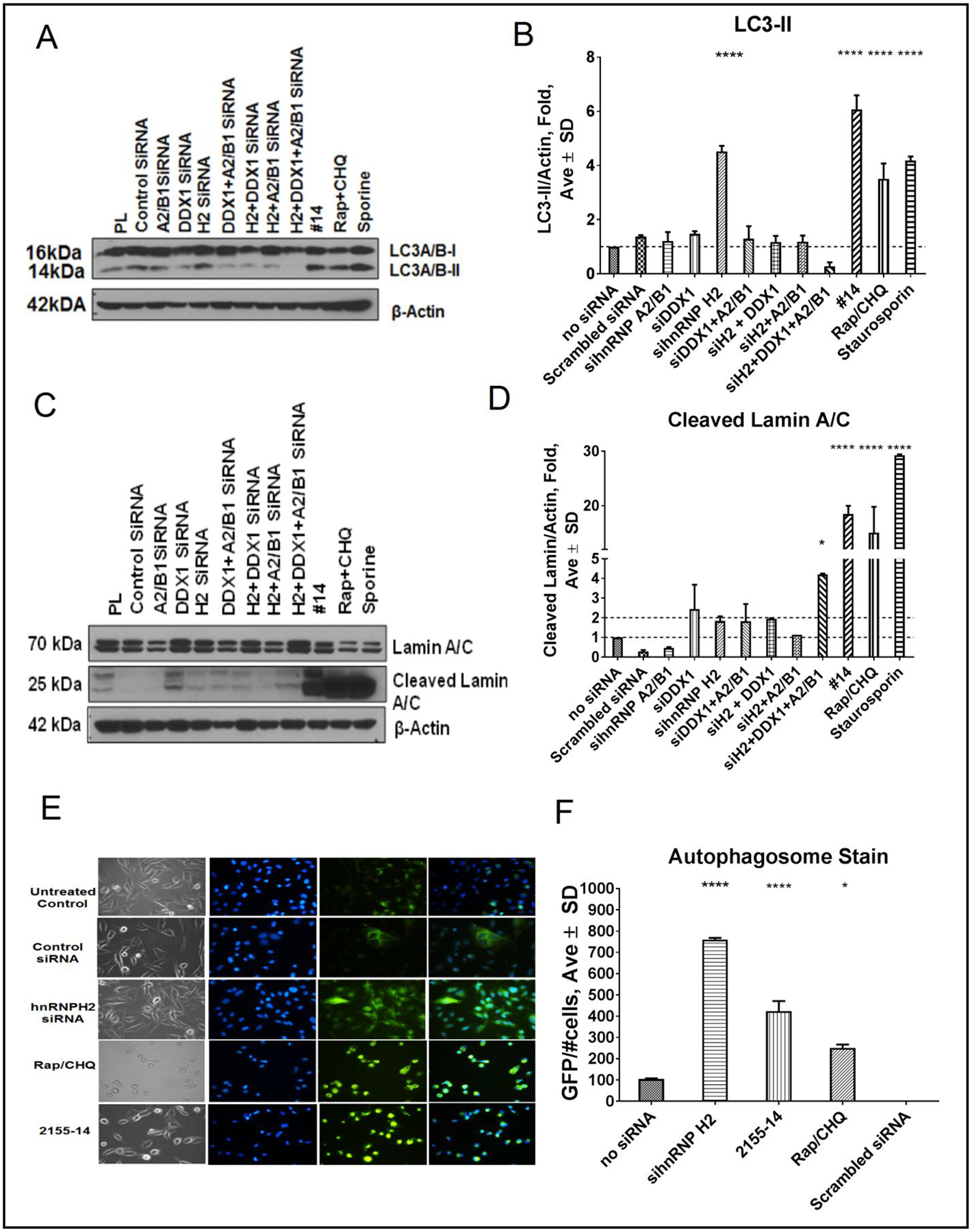
Effect of siRNA knockdown on LC3-II and lamin A/C levels. (A) Representative western blot of LC3-II in WM266–4 lysates in response to siRNA treatment and (B) its quantification. (C) Representative western blot of lamin A/C in WM266–4 lysates in response to siRNA treatment and (D) its quantification. (E) WM266–4 cells showed increased autophagosome staining at 24 h after addition of hnRNP H2 siRNA and 2155–14. Nuclei are stained blue. (F) Autophagy (GFP) channel was used to quantify positive WM266–4 cells. Number of cells present in each well was normalized using DAPI-stained nuclei. One-way analysis of variance (ANOVA) was used followed by Sidak multiple comparisons test. The data shown were the mean ± SD, n=3. ***** - p<0.0001, *** - p<0.001, ** - p<0.01, * - p<0.05, rest = no significance.

To determine if genomic modulation of DDX1, hnRNP H2, and hnRNP A2/B1 recapitulated the effects of administration of 2155–14, we conducted siRNA knockdown experiments. The concentration of siRNAs necessary to achieve complete knockdown was found to be 25 nM for DDX1 and hnRNP H2 and 50 nM for hnRNP A2/B1 (Supplementary Fig. 3). WM266–4 melanoma cells were transfected with above mentioned siRNA concentrations (Fig. 9B) and the effect of knockdown on cell viability, LC3-II, and cleaved lamin A/C levels determined. After 24 h of transfection there was no significant effect on cell viability (Fig. 9C), while at 48 and 72 h the melanoma cells, in the presence of all three siRNAs and combinations thereof, were only ~25% viable as compared to untreated control cells (Fig. 9D and E).This gradual loss of viability was reminiscent of a similar effect in the presence of 2155–14 (Fig. 9F) and thus siRNA treatment appeared to mimic the effects of administration of 2155–14. Surprisingly, only hnRNP H2 siRNA resulted in an increase of LC3-II levels (Fig. 10A and B), suggesting that binding of 2155–14 to hnRNP H2, but not hnRNP A2/B1 or DDX1, leads to the potentiation of autophagy. Levels of cleaved lamin A/C were statistically significantly increased only in the presence of combination of all three siRNAs (Fig. 10C and D), suggesting that binding to all three target proteins by 2155–14 results in the lamin A/C cleavage. Additionally, WM266–4 cells treated with hnRNP H2 siRNA stained positive at levels similar to 2155–14 when autophagosome dye was used (Fig. 10E and F), further indicating that hnRNP H2 modulation alone can potentially lead to autophagy induction.

**Fig. 11.**
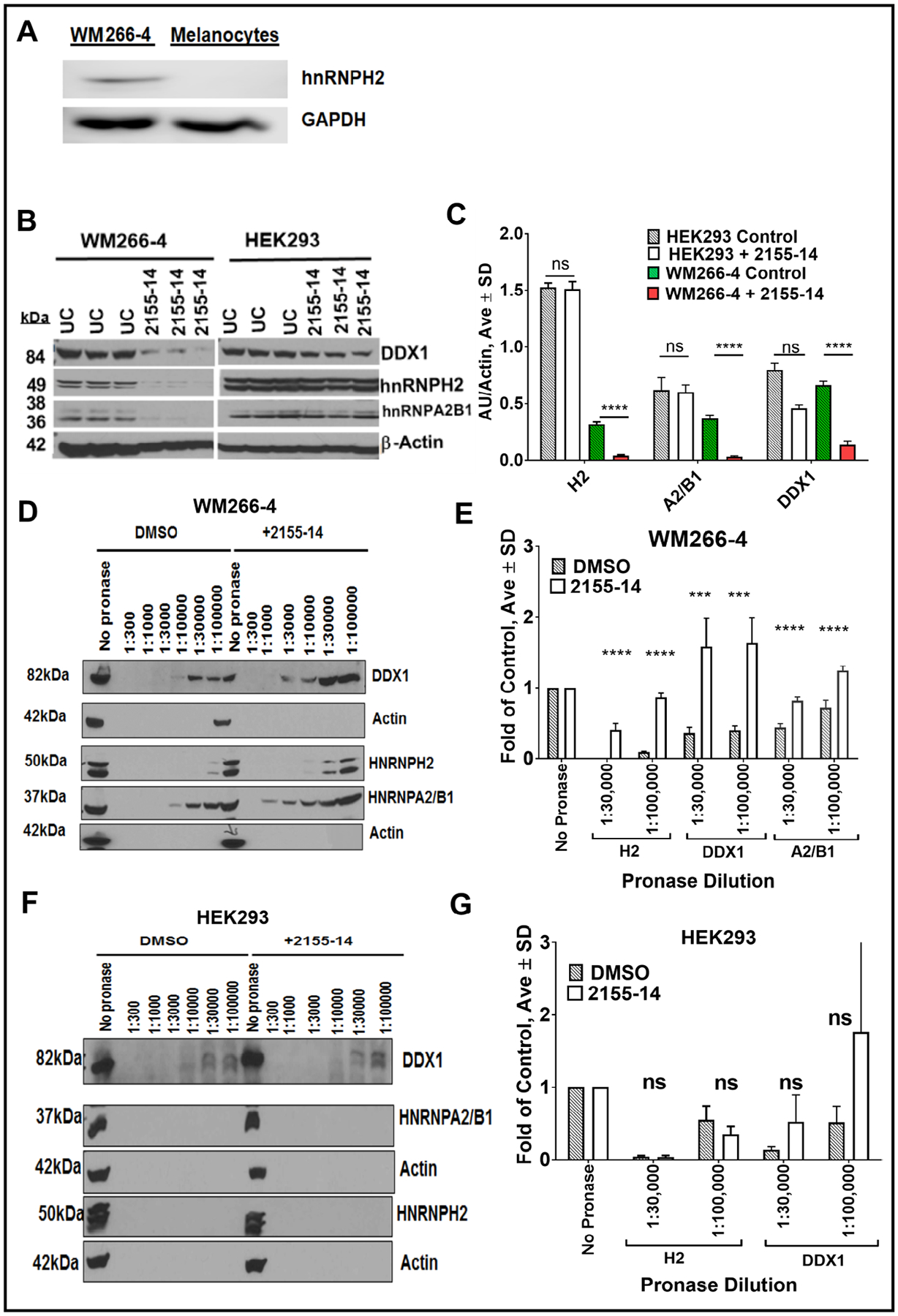
Confirmation of DDX1, hnRNP H2, and hnRNP A2/B1 as targets of 2155–14. (A) Western blot of hnRNP H2 in WM266–4 melanoma cells and melanocytes. (B) Western blot of WM266–4 melanoma and HEK293 cell lysates. Live cells were incubated in presence and absence of 2155–14. (C) Quantification of western blots for (B). (D) Western blot of DDX1, hnRNP H2, and hnRNP A2/B1 in WM266–4 cell lysates after digestion with pronase in the presence and absence of 2155–14. (E) Quantification of western blots for (D). (F) Western blot of DDX1, hnRNP H2, and hnRNP A2/B1 in HEK293 cell lysates after digestion with pronase in the presence and absence of 2155–14. (E) Quantification of western blots for (F). One-way analysis of variance (ANOVA) was used followed by Dunnett post hoc test. The data shown were the mean ± SD, n=3. ***** - p<0.0001, *** - p<0.001, ** - p<0.01, * - p<0.05, rest = no significance.

To initially evaluate if differential cell permeability is the reason 2155–14 is selective for melanoma, we compared the effect of 2155–14 on levels of target proteins in WM266–4 melanoma cells and adult primary melanocytes by incubating 2155–14 with live cells. We did not find hnRNP H2 expression in melanocytes by western blot (Fig. 11A), and thus used human embryonic kidney 293 (HEK293) cells for further comparisons. Following incubation with 2155–14, DDX1, hnRNP H2, and hnRNP A2/B1 levels decreased in WM266–4 melanoma cells but not HEK293 cells (Fig. 11B and C).

To further examine the role of cell permeability on 2155–14 targeting of proteins, we conducted a DARTS (drug affinity responsive target stability) [37, 38] experiment whereby we digested WM266–4 and HEK293 lysates using pronase ± 2155–14 and probed with antibodies for hnRNP H2, hnRNP A2/B1, and DDX1. In lysate fractions treated with 2155–14, all tested proteins were found at higher concentrations in melanoma cells (Fig. 11D and E) compared with HEK293 cells (Fig. 11F and G), suggesting that 2155–14 better protected melanoma proteins from proteolysis. This confirmed that 2155–14 interacts with all three proteins in melanoma cells. Lack of proteolysis protection of proteins from HEK293 cells showed that 2155–14 did not bind target proteins in HEK293 cells. Since 2155–14 protected proteins in cell lysates, structural and/or abundance differences and not cell permeability is the basis for 2155–14 selectivity for melanoma cell proteins. Even though the hnRNP H2 level was significantly higher in HEK293 cells compared with WM266–4 melanoma cells (Fig. 11B and C, compare controls), 2155–14 could not protect it from proteolysis suggesting that selectivity of 2155–14 might not be due to hnRNP H2 abundance but structural differences of the protein in the respective cell lines.

### Probing molecular effectors of cell death with inhibitors

**Fig. 12.**
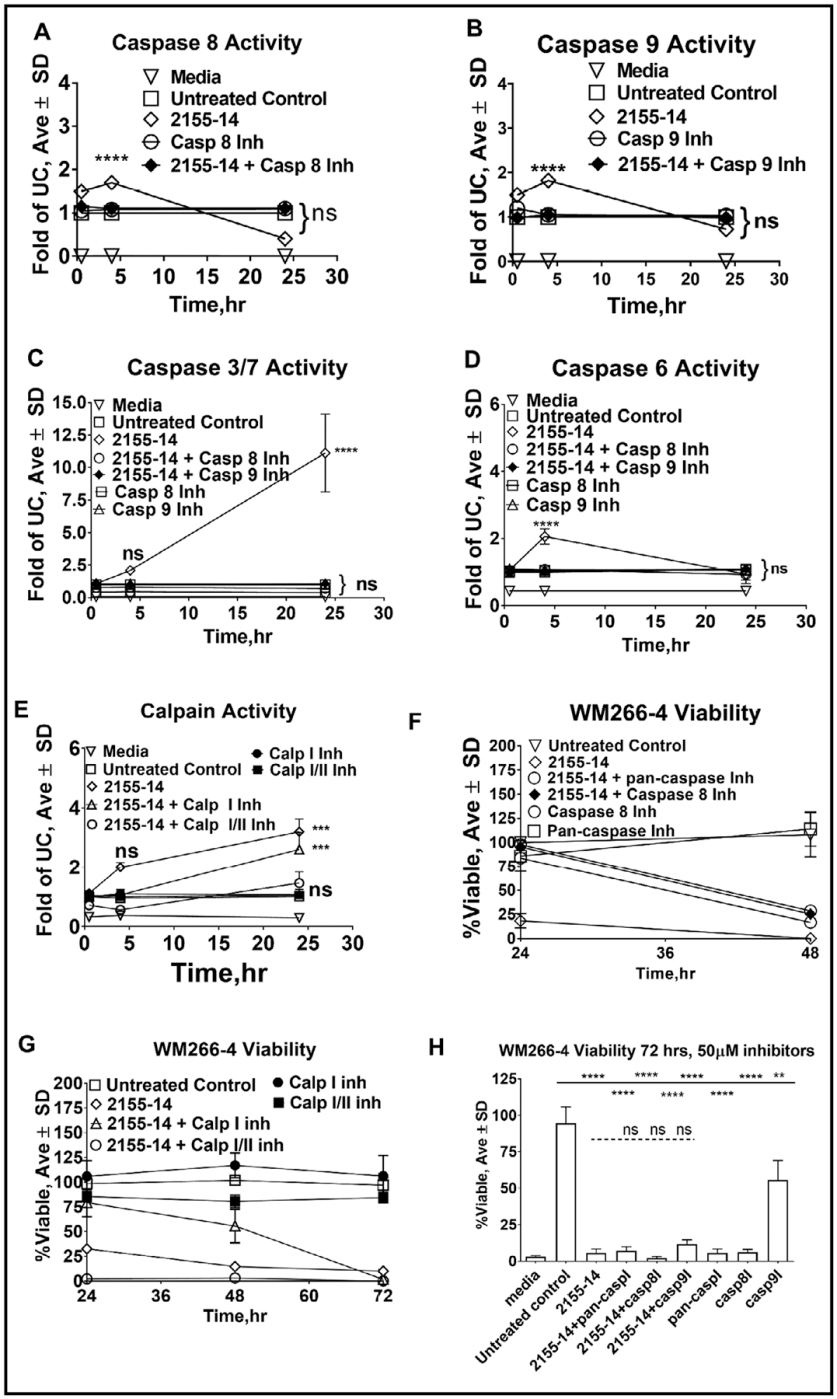
Effect of 2155–14 on caspase and calpain activity and its inhibition on viability of WM266–4 cells. (A) Caspase 8 activity assay results. (B) Caspase 9 activity assay results. (C) Caspase 3/7 activity assay results. (D) Caspase 6 activity assay results. (E) Calpain I/II activity assay results. (F) Cell viability time course after pre-treatment with 10 μM caspase inhibitors. (G) Cell viability time course after pretreatment with 25 μM calpain inhibitors. (H) Cell viability 72 h after pre-treatment with 50 μM caspase inhibitors. One-way analysis of variance (ANOVA) was used followed by Dunnett post hoc test. The data shown were the mean ± SD, n=6. ***** - p<0.0001, *** - p<0.001, ** - p<0.01, * - p<0.05, ns – no significance; 2155–14 was tested at 100 μM.

Compound 2155–14 exerts its effect on melanoma cell viability by inducing ER stress, which, hypothetically, triggers autophagy and loss of mitochondrial membrane potential. Both autophagy and mitochondrial membrane potential loss can lead to cell death via variety of mechanisms. We analyzed activities of enzymes important in the regulation of cell death, calpains and caspases, at 30 min, 4 h, and 24 h after addition of 2155–14. Initiator caspases 8 and 9 exhibited activity in WM266–4 cells 4 h post treatment (Fig. 12A and B), while executioner caspases 6 and 3/7 showed activity at 4 and 24 h, respectively (Fig. 12C and D). Calpains I/II showed ~2-fold increase of activity at 4 h, but this was not statistically significant (Fig. 12E). At 24 h calpains I/II activity reached significance (Fig. 12E). Calpains I/II activity was completely inhibited by a broad-spectrum calpains I/II inhibitor (MDL-28170) and partially inhibited by the selective calpain I inhibitor PD151746 (Fig. 12E), suggesting that both calpain I and II activity was increased in response to 2155–14 treatment. Caspase 3/7 activity was completely inhibited by either the selective caspase 8 inhibitor Z-IETD-FMK or the selective caspase 9 inhibitor Ac-LEHD-CHO (Fig. 12C) and partially inhibited by calpains I/II inhibitor (data not shown) [39–41] suggesting that the activity of caspase 8, caspase 9, and calpains I/II lead to activation of caspase 3/7 by independent pathways. Caspase 8 has been shown to disrupt calpstatincalpain inhibitory complex [42] which suggests that caspase 8 activates both caspase 3/7 and calpains. Caspase 6 activity was completely abrogated by caspase 8 and 9 inhibitors, Z-IETDFMK and Ac-LEHD-CHO, respectively (Fig. 12D), suggesting that caspase 6 is downstream of caspase 8 and 9. Caspase 9 inhibitor Ac-LEHD-CHO was postulated to inhibit caspase 8 as well; therefore, caspase 6 is possibly inhibited due to the caspase 8 inhibitory activity of caspase 9 inhibitor.

To determine whether the inhibition of calpains and caspases has a protective effect on 2155–14-mediated cell viability loss, we pre-incubated WM266–4 cells with abovementioned inhibitors. 24 h after addition of 2155–14, 10 μM pan-caspase inhibitor (Z-VADFMK), 10 μM caspase 8 inhibitor (Z-IETD-FMK), and 25 μM calpain I selective inhibitor (PD151746) restored WM266–4 cell viability to the level of the untreated control (Fig. 12F and G) while 25 μM broad spectrum calpain I/II inhibitor (MDL-28170) further decreased cell viability (Fig. 12G). After 48 h melanoma cells in the presence of 2155–14 alone or in combination with the pan-caspase inhibitor, caspase 8 inhibitor, or and calpain I selective inhibitor were 0%, 20%, 25% and 50% viable, respectively, suggesting that viability cannot be completely rescued by the inhibition of caspases and calpains (Fig. 12F and G). We increased dose of caspase inhibitors to 50 μM and assay time to 72 h to determine if the lack of viability rescue was due to sub-optimal inhibitor concentrations or time of assay. 50 μM pan-caspase inhibitor or caspase 8 inhibitor completely inhibited cell viability (Fig. 12H), making determination of their effect in combination with 2155–14 not possible. 50 μM caspase 9 inhibitor alone decreased viability of WM266–4 cells to 55%. Neither caspase 9 inhibitor nor calpain inhibitors were able to negate the effect of 2155–14 on cell viability.

**Fig. 13.**
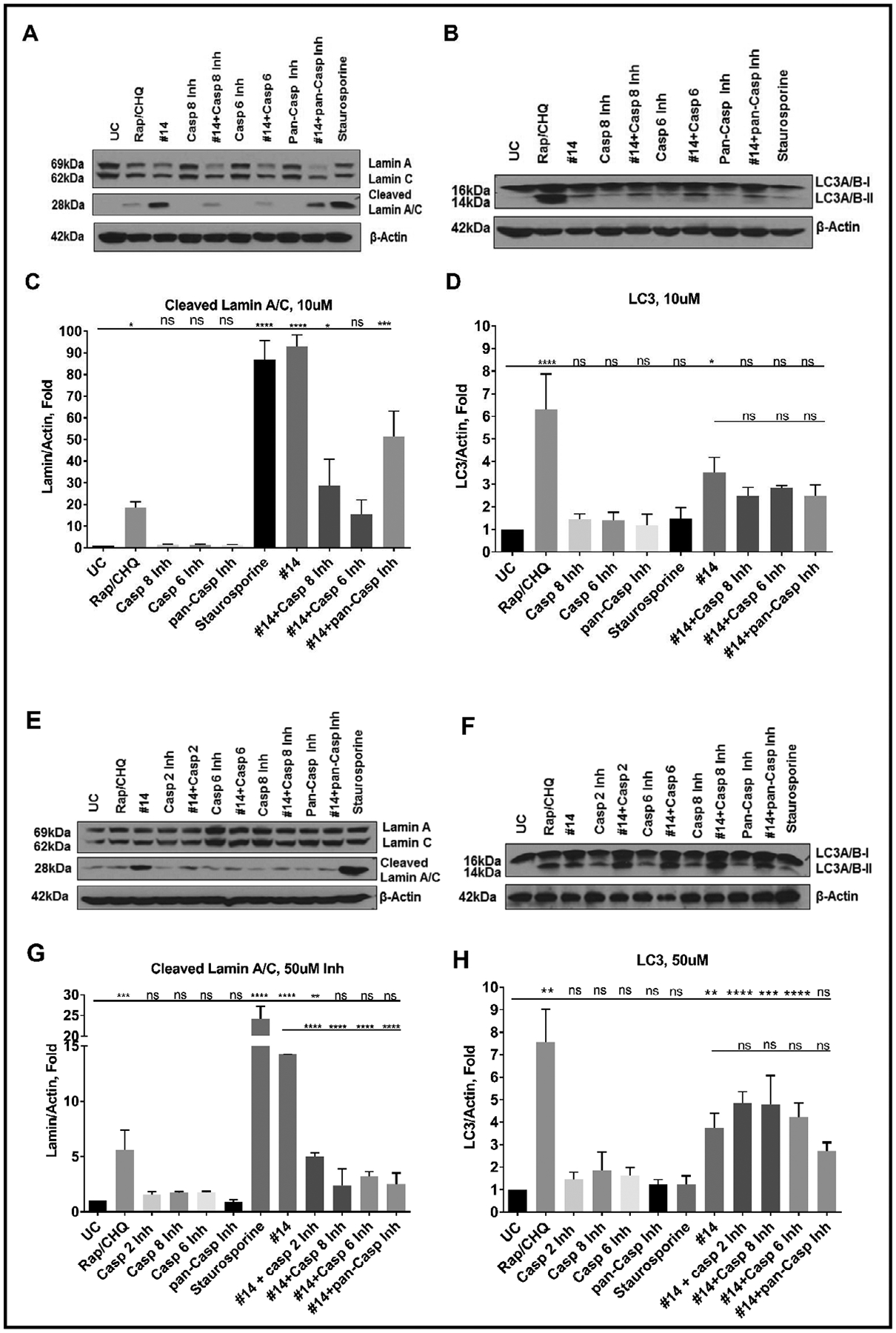
Effect of pretreatment of WM266–4 cells with caspase inibitors on levels of LC3-II and lamin A/C cleavage in the presence of 2155–14. (A) Representative western blot of lamin A/C in the presence of 10 μM caspase inibitors. (B) Representative western blot of LC3 in the presence of 10 μM caspase inibitors. (C) Quantification of western blot of cleaved lamin A/C in the presence of 10 μM caspase inibitors. (D) Quantification of western blot of LC3-II in the presence of 10 μM caspase inibitors. (E) Representative western blot of lamin A/C in the presence of 50 μM caspase inibitors. (F) Representative western blot of LC3 in the presence of 50 μM caspase inibitors. (G) Quantification of western blot of cleaved lamin A/C in the presence of 50 μM caspase inibitors. (H) Quantification of western blot of LC3-II in the presence of 50 μM caspase inibitors. One-way analysis of variance (ANOVA) was used followed by Dunnett post hoc test. The data shown were the mean ± SD, n=3. ***** - p<0.0001, *** - p<0.001, ** - p<0.01, * - p<0.05, ns - no significance. 2155–14 was tested at 100 μM. Rap/CHQ = Rapamycin (5 μM)/Chloroquine (10 μM). Caspase 2 inh = Z-VDVAD-FMK, Caspase 6 inh = Z-VEID-FMK, Caspase 8 inh = Z-IETD-FMK, pan-Caspase inh = Z-VAD-FMK.

Caspases have been shown to play an important role in the crosstalk between apoptosis and autophagy whereby caspase 8 activity is believed to inhibit autophagy and direct cell death to the apoptotic pathways, while the opposite is believed to be true for caspase 9 [43, 44]. Knockdown of caspase 2 increased autophagy in mouse embryonic fibroblasts [45, 46]. In light of the caspase inhibitors failure to rescue viability of WM266–4 cells (Fig. 12), we examined the effect of caspase inhibition on autophagy progress. The autophagy signal was strongest at 24 h in the staining experiment (Fig. 2), and thus we focused on this time point. Initially we pre-treated WM266–4 cells with 10 μM of caspase 8, caspase 6, or pan-caspase inhibitors for 3 h before adding 100 μM 2155–14. Cleavage of lamin A/C by caspase 6 [47] is one of the hallmarks of apoptosis, so we chose to monitor the effect of 2155–14 on levels of cleaved lamin A/C as well as levels of LC3-II to determine whether autophagy resulted in apoptotic cell death. We observed only partial inhibition of lamin A/C cleavage (Fig. 13A and C) which explains why viability (Fig. 12) and apoptosis (Fig. 1) were not rescued by 10 μM caspase inhibitors. Additionally, there was no significant difference in LC3-II levels between cells pre-treated with 10 μM caspase inhibitors and 2155–14 and 2155–14 alone or pre-treated with caspase inhibitors and untreated control (Fig. 13B and D). Therefore, we pre-treated WM266–4 cells with 50 μM of caspase 2, caspase 8, caspase 6, or pan-caspase inhibitor. Cleavage of lamin A/C was almost completely abrogated by 50 μM of caspase-8, caspase 6, or pan-caspase inhibitor, and, to the lesser extent, caspase 2 inhibitor (Fig. 13E and G). However, there was no significant difference in LC3-II levels between cells pretreated with caspase inhibitors and 2155–14 and 2155–14 alone and a significant difference between cells pre-treated with caspase inhibitors and untreated control (Fig. 13F and H).

**Fig. 14.**
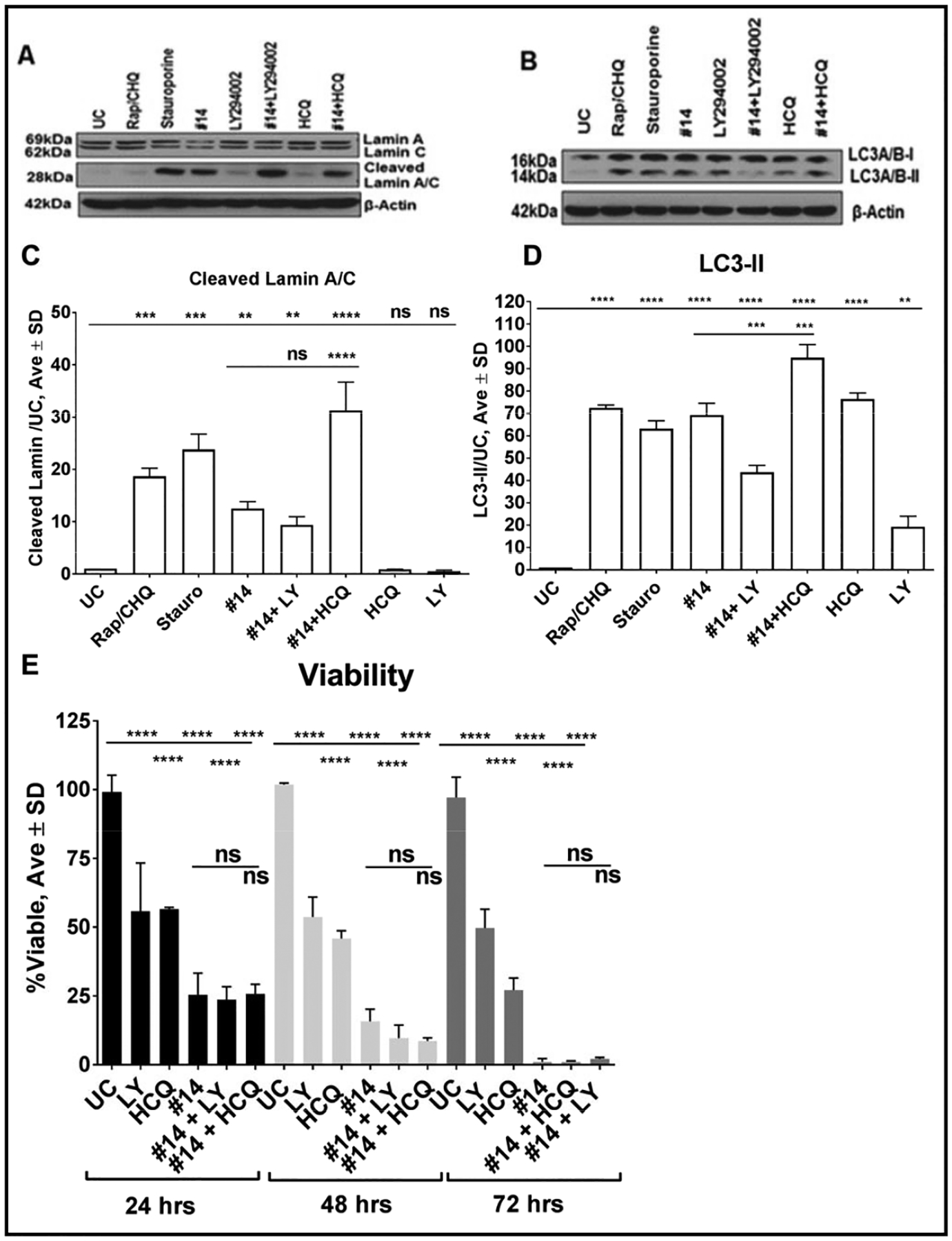
Effect of pre-treatment of WM266–4 cells with autophagy inhibitors on viability and levels of LC3-II and lamin A/C cleavage in the presence of 2155–14. (A) Representative western blot of lamin A/C. (B) Representative western blot of LC3. (C) Quantification of western blot of lamin A/C. 2155–14 was tested at 100 μM, LY and hydroxychloroquine (HCQ) were tested at 10 μM. (D) Quantification of western blot of LC3. The data shown are the mean ± SD, n=3. (E) Viability of WM266–4 cells after pre-treatment with autophagy inibitors in the presence of 2155–14. The data shown were the mean ± SD, n=6. One-way analysis of variance (ANOVA) was used followed by Dunnett post hoc test. ***** - p<0.0001, *** - p<0.001, ** - p<0.01, * - p<0.05, ns - no significance.

Caspase and calpain inhibition experiments showed a lack of caspase control over autophagy and cell death induced by 2155–14. We examined whether modulation of autophagy would have an effect on apoptosis and cell viability. Western blot analysis of cells treated with 2155–14 showed that pre-treatment with autophagy inhibitor LY294002 (a PI3K inhibitor) had no effect on cleaved lamin A/C levels (Fig. 14A and C) suggesting a lack of apoptosis inhibition. LY294002 alone increased LC3-II levels 20-fold as compared to untreated control fold (Fig. 14B and D) and while a combination of LY294002 and 2155–14 showed a significant decrease of LC3-II as compared to 2155–14 alone it was still ~40-fold greater than in the untreated control, which resulted in the loss of cell viability comparable to 2155–14 alone (Fig. 14E). This suggested that 2155–14 is capable of overriding or bypassing PI3K inhibition leading to the cell death. Additionally, the opposite effect of LY294002 on baseline and ER stress-induced autophagy indicated a different role of PI3K signaling in these processes in melanoma.

Hydroxychloroquine (HCQ), a lysosomal lumen alkalizer and an inhibitor of an autophagic flux [48], pre-treatment increased levels of both LC3-II and cleaved lamin A/C (Fig. 14A–D), consistent with the failure of HCQ to rescue the viability of WM266–4 melanoma cells treated with 2155–14 (Fig. 14E). Since autophagy could be used by melanoma cells as a survival mechanism [49, 50] its inhibition could synergize with the effect of 2155–14 suggesting a possibility of a combination therapy.

**Fig. 15.**
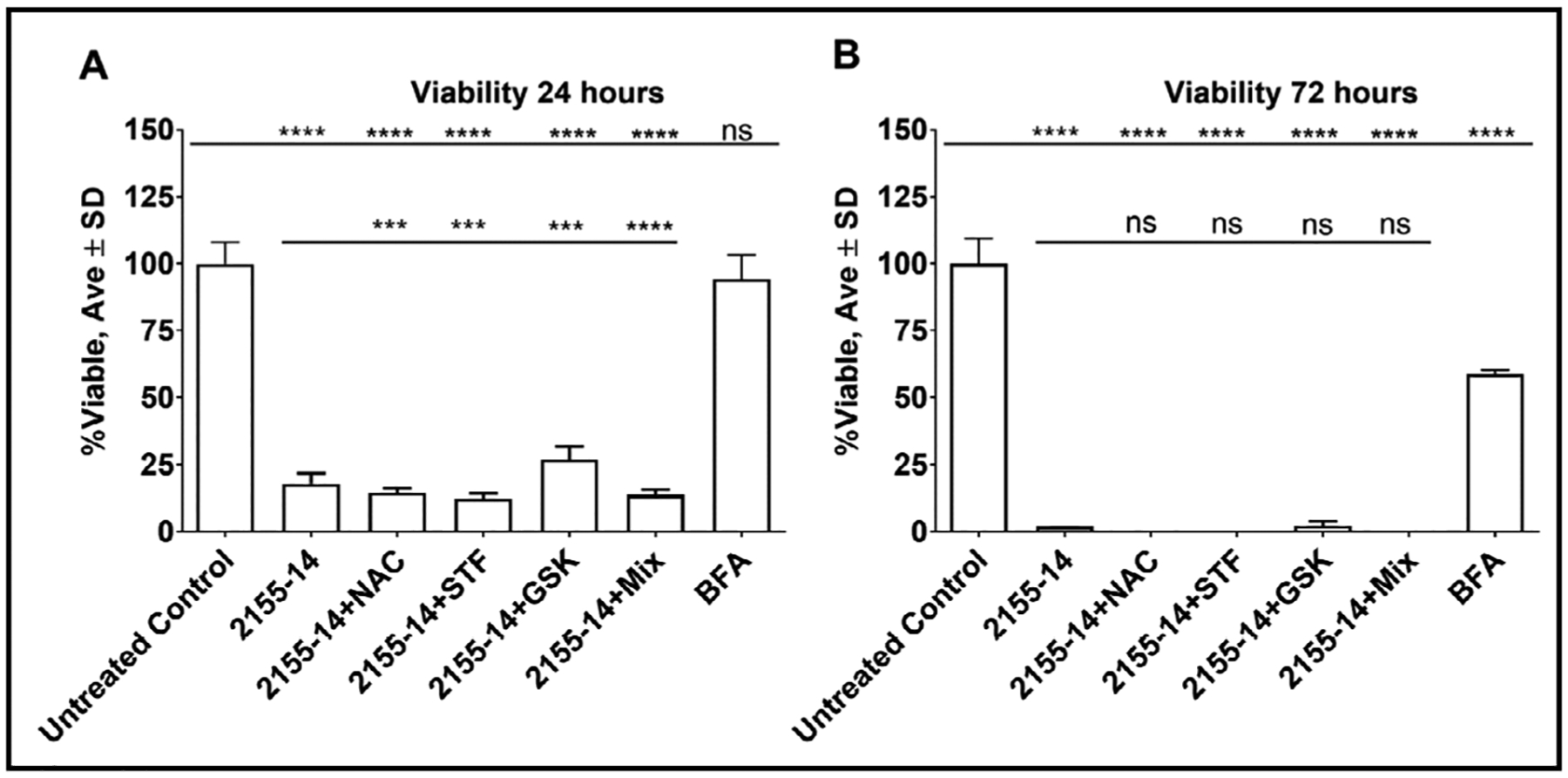
Effect of pretreatment of WM266–4 cells with ER stress inhibitors on viability in the presence of 2155–14. (A) WM266–4 cell viability 24 h after 2155–14 addition. (B) WM266–4 cell viability 72 h after 2155–14 addition. 2155–14 was tested at 100 μM, STF-83010 (STF) was tested at 60 μM, N-acetyl cysteine (NAC) was tested at 10 μM, and GSK2606414 (GSK) was tested at 1 μM. The data shown were the mean ± SD, n=6. One-way analysis of variance (ANOVA) was used followed by Dunnett post hoc test. ***** -p<0.0001, *** - p<0.001, ** - p<0.01, * - p<0.05, ns - no significance.

We lastly examined the effect of ER stress pathway inhibition on melanoma cell viability. Several inhibitors of the ER stress pathway were utilized, including N-acetyl cysteine (NAC, a polypharmacological agent known to interfere with PERK, IRE1, and ATF6 branches of the ER stress pathway) [35], STF-83010 (an IRE1 RNase activity inhibitor) [35], and GSK2606414 (a PERK autophosphorylation inhibitor) [51]. Pre-treatment with ER stress pathway inhibitors alone or in combination had neither protective nor deleterious effects on WM266–4 cell viability (Fig. 15A and B).

## Discussion

In the present study, DDX1, hnRNP H2 (and possibly H1), and hnRNP A2/B1 have been identified as targets of an anti-melanoma compound 2155–14. hnRNP H2, hnRNP H1, and hnRNP A2/B1 are involved in mRNA splicing, export, and stability [52], while helicase DDX1 is involved in mRNA/miRNA [53] and tRNA splicing [54] and is known to interact with another ribonuclear protein, hnRNP K [53]. DDX1, hnRNP H2, and hnRNP A2/B1 are constituents of multi-protein spliceosomal complexes [52, 55]. DDX1 and hnRNP A2/B1 could potentially form a complex with hnRNP H2 and hnRNP H1, explaining co-precipitation of all three spliceosomal proteins with 2529–7. Neither of the identified proteins have been previously connected to the regulation of autophagy. It is possible, however, that their splicing targets may be involved in autophagy. Database and literature search revealed that hnRNP H1 controls expression of multiple spliceosomal structural elements (e.g., hnRNPA1, hnRNPU, hnRNPC, RBM25), MAPK signaling (e.g., MAP2K7, AKT2 AKT1) and ubiquitin mediated proteolysis (e.g., UBE2K, NEDD4L, UBE2J2) [56]. Additionally, it controls RNA splicing events *via* its targets that functionally participate in splicing (e.g., hnRNPA2/B1, hnRNPH3, hnRNPH1, hnRNPK). Mechanistically, hnRNPK knockdown upregulated basal autophagy *via* modulating the expression level of HDAC6 to influence the autophagosome-lysosome fusion [57]. hnRNPK expression is controlled by hnRNPH1 which could explain why targeting of hnRNPH2 and H1 by both 2155–14 and siRNA leads to autophagy. siRNA knockdown of DDX1 and hnRNPA2/B1 did not result in autophagy suggesting that these two proteins are not involved in the control of autophagy.

hnRNP K, a known binding partner of DDX1 [53], was shown to regulate autophagy and was upregulated in acute myeloid leukemia cells derived from non-remission patients [58]. In this context, an increase of LC3-II levels as the result of genomic modulation of hnRNP H2 is certainly very interesting. hnRNP H2 has never been shown to regulate autophagy; therefore, future studies of its connection to autophagy and potential to drive cell death could lead to a novel approach to melanoma drug discovery. No prior studies have connected the protein targets of 2155–14 to melanoma progression, suggesting they could be novel targets for melanoma drug discovery. Future studies will ascertain which targets or combination of targets can be engaged for drug discovery. Also, to the best of our knowledge, there has been no reports of small molecules that can bind to any of the proteins identified herein, therefore 2155–14 is a “first-in-class” compound. Based on these considerations, individual probes for each protein will need to be developed to assess the effects of binding to a single protein as opposed to binding to a tertiary complex (DDX1/hnRNP A2/B1/hnRNP H2).

While hnRNPH2 and hnRNPH1 have not been considered for cancer drug discovery, there are molecules that bind other spliceosomal components that are being investigated for cancer therapy (reviewed in [59]). Examples of small molecules include herboxidienes, pladenolides and spliceostatins that target SF3B1. This suggests a growing interest for targeting splicing for cancer drug discovery.

hnRNP H1/H2 was shown to be responsible for drug resistance to capecitabine due to aberrant splicing of thymidine phosphorylase mRNA in monocytic/macrophage leukemia cell lines THP-1 and U937 [60]. All of the protein targets identified in the present study participate in multi-component complexes and, therefore, are involved in many processes. For example, numerous oncogenes were shown to be direct targets of hnRNPs, indicating their importance in cancer development [61]. At this stage it is not possible to ascertain the exact process affected by the binding of 2155–14 to these proteins. However, based on the ER stress and autophagy-mediated death of WM266–4 and M14 melanoma cells it is likely that binding of 2155–14 results in interference with protein translation which is known to induce autophagy in melanoma [62].

Mechanistically, binding of quercetin to hnRNP A1 was shown to prevent its shuttling between the nucleus and cytoplasm leading to apoptotic cell death of PC-3 cells with 100 μM IC_50_ [63]. A nanoparticle-conjugated aptamer specific to hnRNP A2/B1 was able to inhibit proliferation of multiple cancer cell lines [64]. This evidence in combination with our results suggests 2155–14 represents a lead for future optimization studies targeting DDX1, hnRNP H2, and hnRNP A2/B1 binding.

Both siRNA and 2155–14 treatment resulted in a gradual time-dependent loss of viability consistent with late onset activation of caspase 3/7 at 24 h after 2155–14 addition. Modulation of nuclear proteins could generally lead to slower cell death, as compared to fast apoptosis-inducing drugs, which could allow for autophagy to take its course resulting in degradation of intracellular proteins and organelles. This, in turn, could lead to smaller amounts of intracellular proteins being released into the patients’ bloodstream upon cell death resulting in fewer immunogenic effects.

Although 2155–14 was utilized at relatively high concentrations in the present study, the activity of the compound towards melanoma cells was as good or better than the FDA approved compound vemurafenib (Table 2). Importantly, while binding to multiple molecular targets, 2155–14 exhibited selectivity for melanoma cell lines while sparing multiple other cancer and non-malignant cell lines. In case of melanocytes, the lack of effect on viability can be explained by the surprising lack of expression of hnRNP H2 (Fig. 11A). hnRNP A2/B1 was found to be overexpressed in G361 melanoma cells as compared to melanocytes [65]. In yet another example of potential selectivity of spliceosomal modulation, binding of hnRNP A2/B1 and HSP90 by the natural product phenanthrene-based tylophorine derivative-1 (PBT-1) had potent activity against lung cancer and no overt toxicity [66] suggesting that binding of multiple targets in cancer cells can be well tolerated. Moreover, it was proposed that polypharmacology of anti-cancer agents can be advantageous in preventing of drug resistance [67, 68] common for single or even dual target melanoma drugs [9, 11–13]. The mechanisms of mRNA processing are believed to be well-conserved between different cell types and it is not immediately clear why 2155–14 does not inhibit viability of nonmelanoma cancer cells or non-malignant cells. To inhibit melanoma viability, 2155–14 appears to need to bind directly to hnRNP H2 and either DDX1 or hnRNP A2/B1. This hypothesis is supported by our data demonstrating that 2529–3, which binds to just hnRNP H2, does not affect melanoma cell viability as efficaciously as 2529–7, which pulls down the three different proteins (Fig. 7A, 8, and 9A). hnRNP H2 may have a unique isoform or post-translational modification in melanoma, which would account for the ability of 2155–14 to protect it against pronase digestion but not hnRNP H2 from HEK293 cells (Fig. 11). This would be reminiscent of the cancer-associated isoform of proliferating cell nuclear antigen (PCNA). A peptide derived from the unique region of cancer-associated PCNA was cytotoxic towards cancer cells but considerably less so towards normal cells [69–71]. Alternatively, 2155–14 binding to two proteins directly may position one protein to block pronase cleavage of hnRNP H2.Further investigation of the molecular basis of 2155–15 selectivity can improve our understanding of melanoma cell biology and lead to the drugs with decreased toxicity as compared to the existing therapies.

Compound 2155–14 induces ER stress leading to the potentiation of basal autophagy and melanoma cell death in BRAF and NRAS mutated melanoma cells indicating that this can be a novel approach to a much-needed broad-spectrum melanoma therapy. Autophagy plays a role in the development as well as overcoming drug resistance in melanoma ^[72]^. hnRNP H2 modulation may also address the lack of therapies for NRAS mutants and other subtypes of melanoma. Autophagy regulates cytokine secretion and antigen presentation and, therefore, contributes to anti-tumor immune responses [73–75].

The precise mechanism by which increased autophagy progresses to cell death has not been investigated in melanoma. ER stress, autophagy, calpain, and caspase inhibitors failed to prevent loss of viability of WM266–4 cells, and therefore it is not clear whether cells treated with 2155–14 die *via* apoptotic or autophagic pathway. Caspase inhibitors were able to completely inhibit cleavage of lamin A/C, but not the increase of LC3-II and did not rescue cell viability suggesting that caspase activity might not be necessary to drive 2155–14-mediated cell death. Interestingly, a biotinylated analog of 2155–14, 2529–7, did not induce cleavage of lamin A/C, but increased levels of LC3-II leading to the WM266–4 cell death with potency similar to 2155–14 (IC_50_ = 4 ± 0.5 and 3.3 ± 0.5 μM for 2155–14 and 2529–7, respectively), supporting the hypothesis that both apoptotic and autophagic cell death mechanisms are enacted. Cell viability loss was only slowed down by calpain μ-selective inhibitor PD151746 suggesting its role in the regulation of autophagy-driven cell death. Colunga et al. [76] showed that calpain activation in melanoma 3D culture can lead to inhibition of growth which was restored by calpain-specific inhibitor PD150606 but not by pan-caspase inhibitor Z-VAD-FMK, which is in agreement with our data. Interestingly, PD150606 also restored expression of p62, a marker of autophagy, suggesting a complex role of calpain in regulation of autophagy in melanoma. Additionally, calpains were shown to affect mitochondrial membrane potential [77], which could explain the effect of 2155–14 on mitochondrial potential. Inhibition of calpain μ activity was overcome after 24 h of co-application with 2155–14, which lead to the time-dependent loss of viability. This could be due to either a dose-limiting effect of calpain μ inhibitor PD150606 or its inability to completely block the processes initiated by 2155–14. Overall, it appears that apoptosis caused by 2155–14 is initiated by caspase 8 *via* activating calpains and executed by caspase 3/7. However, the inability of calpain inhibitors to prevent cell death suggests that apoptosis is not the only cause of loss of cell viability.

The inability of autophagy, caspase, and calpain inhibitors to prevent cell death after exposure to 2155–14 suggests a contribution of another type of cell death different from autophagy-driven caspase-dependent apoptosis, such as autosis. Autosis, an autophagic cell death due to excessive uncontrollable autophagy [78–80], has no known morphological or molecular markers and is a poorly studied controversial subject [81]. However, autosis cannot be completely discarded at this stage. Our data showing that the biotinylated analog of 2155–14, 2529–7, induces autophagy and causes cell death in the absence of cleavage of lamin A/C, supports this hypothesis.

**Fig. 16.**
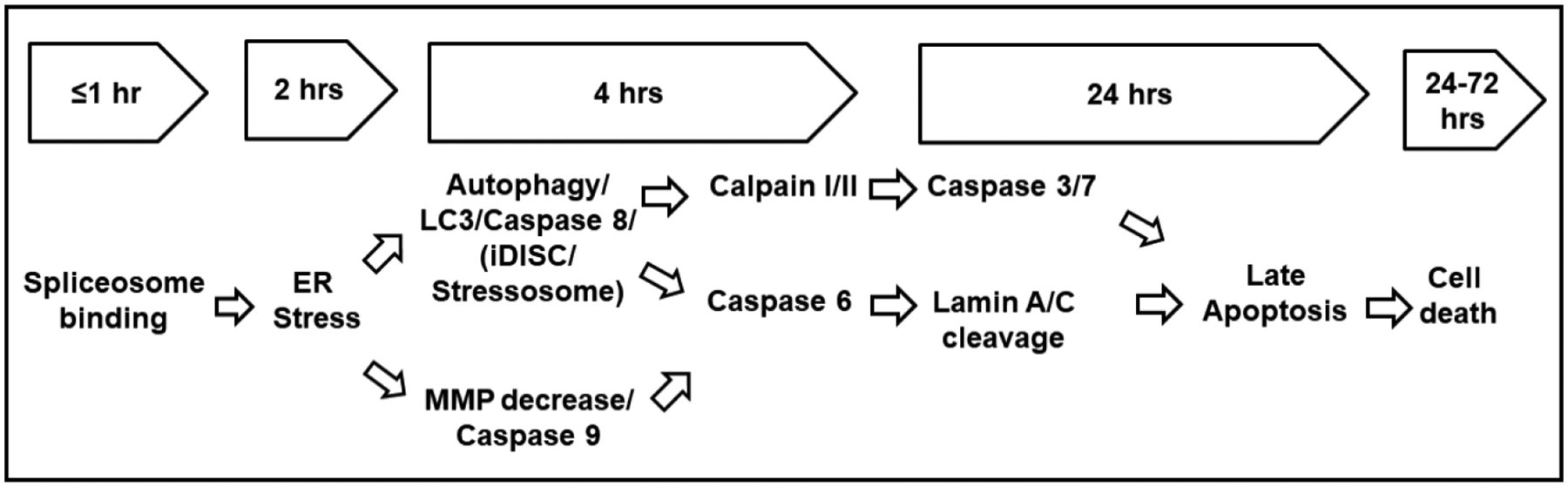
Proposed mechanism of action of 2155–14.

We can develop a mechanistic hypothesis for the action of compound 2155–14 (Fig. 16). Binding of 2155–14 to the spliceosome within 1 h (Fig. 8C) leads to ER stress (Fig. 5, detected 2 h after 2155–14 treatment). 4 h after 2155–14 treatment, ER stress activates both mitochondrial and extrinsic apoptotic pathways and autophagy [33] (supported by caspase 9 and 8 activities (Fig. 12A and B), autophagosomal staining (Fig. 2B), and a decrease in mitochondrial membrane potential (Fig. 3C and D)). Caspase 8 and 9 activity leads to the activation of caspase 6 (Fig. 12D) and possibly calpains 4 h after 2155–14 addition (Fig. 12E). Calpains activity 24 h after 2155–14 addition leads to caspase 3/7 activation (Fig. 12C) while caspase 6 activity leads to lamin A/C cleavage (Fig. 13A, C, E, and G). Both caspase 3/7 and caspase 6 activity lead to apoptosis initiation 24 h after 2155–14 addition and cell death. Delay in apoptosis detection until 24 h after 2155–14 treatment could be explained by the protective role of autophagy, which is supported by lack of viability rescue by autophagy inhibitors (Fig. 14).

Further in-depth studies are needed to dissect the mechanism of 2155–14 action. Of particular interest are the mechanistic details of ER stress induction after binding of 2155–14 to the spliceosome, which could advance our understanding of processes controlled by hnRNP H2.

## Conclusion

In the present study, we have identified potential molecular targets of novel spliceosomal probe 2155–14 in melanoma cells – DDX1, hnRNPH2 and hnRNPA2/B1. Our data suggest that binding to all three of these proteins may be necessary to have an effect on melanoma cell viability. Further studies are needed to determine exact mode of interaction of 2155–14 with DDX1, hnRNPH2 and hnRNPA2/B1. Additionally, we have demonstrated that 2155–14 mechanism of action is mediated by ER stress leading to autophagy and apoptotic cell death suggesting that modulation of spliceosomal proteins may represent a novel approach to melanoma therapy.

## Supplementary Material

Supplemental Material

## Figures and Tables

**Table 1.** Cell lines used in the present studies

Cell line	Mutation	Stage	Human melanoma subtype [6]
WM115	^V600D^BRAF/^−/−^PTEN [82]	Primary	1.2
WM266-4	^V600D^BRAF/^−/−^PTEN [82]	Metastatic	1.2
SK-MEL-28	^V600E^BRAF/^R24C^CDK4[30, 82]	Primary	1.4
M14	^G12C^NRAS ^[83]^	Metastatic	4.1
SK-MEL-2	^Q12R^NRAS [82]	Metastatic	4.1
B16F10	p53/PTEN	Metastatic	N/A

**Table 2.** Effect of 2155–14, 2155–18, and vemurafenib on viability of human melanoma cells and melanocytes and murine melanoma cells and fibroblasts. All results are IC_50_’, μM (n=3). NT – not tested, M – male, F – female. M14 - ^G12C^NRAS metastatic, WM266–4 - ^V600D^BRAF/^−/−^PTENmetastatic, SKMEL28 - ^V600E^BRAF/^R24C^CDK4 primary, WM115 - ^V600D^BRAF/^−/−^PTEN primary. Values reported previously elsewhere are indicated by citation numbers

ID	B16F10	MEF	M14 (M)	SKMEL28 (M)	SKMEL2 (M)	WM115 (F)	WM266–4 (F)	Primary adult melanocytes
2155–14	3±0.2[17]	>100	4±0.3[17]	0.6±0.4[17]	4±0.6	7±1	4±0.5	>100
2155–18	1±0.1[17]	49±3	0.9±0.1[17]	0.8±0.1[17]	4±0.1	5±1	5±1	>100
vemurafenib	NT	25±2	1±0.2	4.6[30]	1.4±0.7	5±0.9	3±0.4	NT

**Table 3.** Effect of 2155–14 and 2155–18 on viability of human cancer and non-malignant cell lines cell lines. All results are IC_50’_, μM (n=3). NT – not tested. Values reported previously elsewhere are indicated by citation numbers. OPC – oligodendrocyte precursor cells

	Cancer				Non-malignant			
ID	A549 (Lung)	HEPG2 (Liver)	MDA-MB-231 (Breast)	U 87 (Brain)	BJ (Skin fibroblast)	HEK293 (Kidney)	CHO-K (Ovary)	OPC (Brain)
2155–14	>100[17]	>100[17]	>100[17]	>100	>100	>100	>100[17]	>10
2155–18	22±2[17]	>100[17]	>100[17]	>100	15±3	30±3	>100[17]	>10

**Table 4.** Results of proteomic analysis of pulldown of lysates of WM266–4 cells with compound 2155–14 and its biotinylated analogs. All units are scores based on unique peptide counts. Top hits for each band are listed. Protein with highest score in each band is in bold font

Band #	1 h	24 h	MW range, kDa	Protein ID	Accession #	Score
1	No	Yes	100–75	ATP-dependent RNA helicase DDX1	Q92499	71.25
				hnRNP U-like protein 1	Q9BUJ2	37.61
				ATP-dependent RNA helicase DDX3X	000571	35.63
2	Yes	Yes	75–50	hnRNP H2	P55795	71.93
				hnRNP HI	P31943	63.06
3	Yes	Yes	37–25	hnRNP A2/B1	P22626	85.5
4	Yes	Yes	37–25	hnRNP A2/B1	P22626	45.77

## Abbreviations

hnRNP: heterogenous nuclear ribonuclear protein
DDX: DEAD-box helicase
CCCP: carbonyl cyanide m-chlorophenyl hydrazine
sXBP1: short X-box binding protein 1
HCQ: hydroxychloroquine
NAC: N-acetyl cysteine
PCNA: proliferating cell nuclear antigen
PBT-1: phenanthrene-based tylophorine derivative-1
HSP90: heat shock protein 90
PERK: PRKR-like endoplasmic reticulum kinase
IRE1: Inositol-requiring protein 1
ATF6: Activating transcription factor 6
CLIP: cross-linking immunoprecipitation
RIP: RNA immunoprecipitation
