# Supplementary material for: A Novel Probe for Spliceosomal Proteins that Induces Autophagy and Death of Melanoma Cells Reveals New Targets for Melanoma Drug Discovery
Source: Cell Physiol Biochem. Author manuscript; Available in PMC 2020 Jan 30. (PMC6990463; doi:10.33594/000000164)

# Supplemental Material

# A Novel Probe for Spliceosomal Proteins that Induces Autophagy and Death of Melanoma Cells Reveals New Targets for Melanoma Drug Discovery

Manikandan Palrasu[a] Anna M. Knapinska[b] Juan Diez[c] Lyndsay Smith[b]
Travis LaVoi[d] Marc Giulianotti[d] Richard A. Houghten[d] Gregg B. Fields[b]
Dmitriy Minond[c,e]

[a]Department of Drug Discovery and Development, Harrison School of Pharmacy, Auburn University, Auburn, AL, USA, [b]Department of Chemistry & Biochemistry, Center for Molecular Biology & Biotechnology, Florida Atlantic University, Jupiter, FL, USA, [c]Rumbaugh-Goodwin Institute for Cancer Research, Nova Southeastern University, Fort Lauderdale, FL, USA, [d]Torrey Pines Institute for Molecular Studies, Port St. Lucie, FL, USA, [e]Dr. Kiran C. Patel College of Allopathic Medicine, Nova Southeastern University, Fort Lauderdale, FL, USA

## Compound purification and characterization.

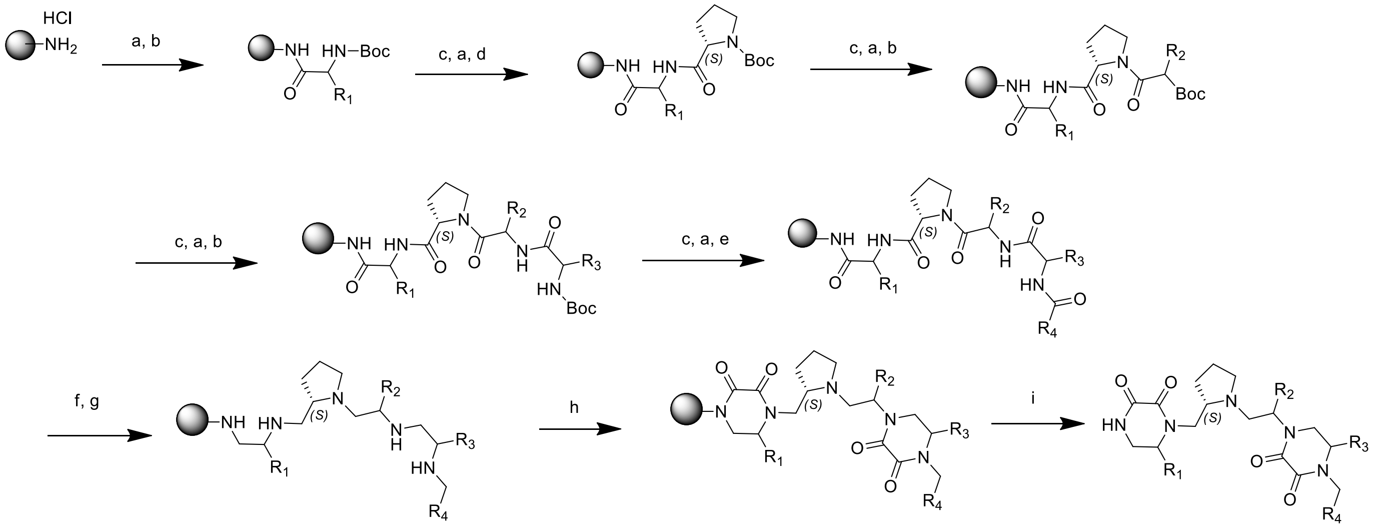


**Supplementary Figure 1A. Synthesis procedure for (S)-1((R)-1((S)-2-(((S)-6-benzyl-2,3-dioxopiperazin-1-yl)methyl)pyrrolidin-1-yl)-3-cyclohexylpropan-2-yl)-5-(4-hydroxybenzyl)-4-phenethylpiperazine-2,3-dione:** (a)5%DIEA/95%DCM, 3x 2min; (b) Boc-AA (6eq), DIC(6eq) ,HOBt (6eq),DMF, 2hr; (c) 55%TFA/45%DCM, 30min; (d) Boc-L-Pro-OH (6eq),DIC (6eq), HOBt (6eq), DMF (6eq), 2hr; (e) RCOOH (10eq), DIC (10eq), HOBt (10eq), DMF, 2hr; (f) BH3/THF(40eq), 65C, 96hr; (g) Piperidine, 65C, 18hr; (h) (COIm)2 (10eq), Anhydrous Atmosphere, 18hr; (i)HF/Anisole, 0C, 1.5hr

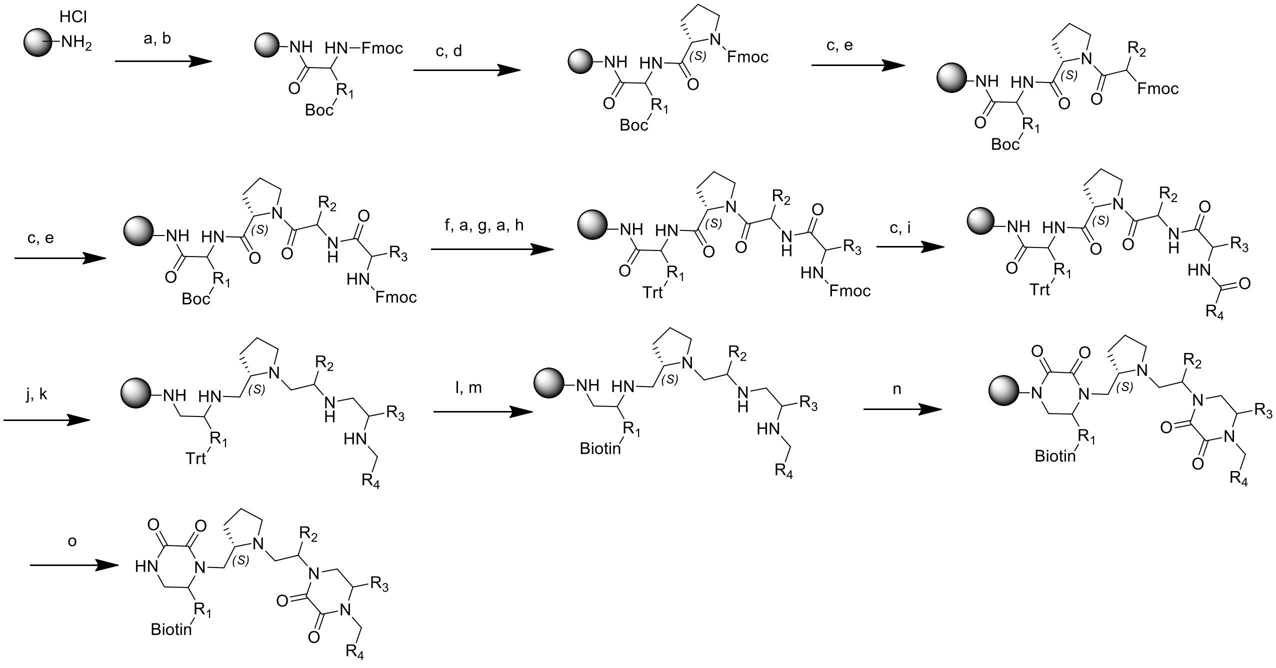


**Supplementary Figure 1B. Synthesis procedure for Biotin tagged pyrrollidine-bis-diketopiperazines used in present studies:** (a)5%DIEA/95%DCM, 3x 2min; (b) Fmoc-AA(Boc) (6eq), DIC(6eq) ,HOBt (6eq),DMF, 2hr; (c) 20%Piperidine/80%DMF, 2x 30min; (d) Fmoc-L-Pro-OH (6eq),DIC (6eq), HOBt (6eq), DMF (6eq), 2hr; (e) Fmoc-AA (6eq), DIC(6eq) ,HOBt (6eq),DMF, 2hr; (f) 55%TFA/45%DCm, 30min; (g) Trt-Cl (5eq), DIEA (10eq), 10%DMF/90%DCM, 2hr; (h) Trt-Cl (5eq), DIEA (10eq), 10%DMF/90%DCM, 2hr; (i) RCOOH (10eq), DIC (10eq), HOBt (10eq), DMF, 2hr; (j) BH3/THF(40eq), 65C, 96hr; (k) Piperidine, 65C, 18hr; (l) 2%TFA/5%TRIS,95%DCM. 3x 2min; (m) Biotin (10eq), DIC (10eq), DMF, 2hr (n) (COIm)2 (10eq), Anhydrous Atmosphere, 18hr; (o)HF/Anisole, 0C, 1.5hr

**(S)-1((R)-1((S)-2-(((S)-6-benzyl-2,3-dioxopiperazin-1-yl)methyl)pyrrolidin-1-yl)-3-cyclohexylpropan-2-yl)-5-(4-hydroxybenzyl)-4-phenethylpiperazine-2,3-dione.** Using General Scheme (scheme 1) for the synthesis of bis-cyclic diketopiperazines compounds 2155-14 and 2529-1 were synthesized using the following reagents: (2 g) MBHA resin starting material, Boc-L-Phenylalanine -OH ($R_1$), Boc-D-Cyclohexylalanine-OH ($R_2$), Boc-L-Tyrosine(2-Br-Z)-OH ($R_3$), and Phenylacetic Acid ($R_4$). The final crude product was purified using HPLC as described above, with a gradient of (B) 0/5, 2/5, 4/20, 40/55. **Isolated Mass** 504.2mg, **%yield** 28.62%.

**$^1$H NMR** (400 MHz, DMSO-$d_6$) δ ppm 0.80 - 1.05 (m, 2 H) 1.08 - 1.20 (m, 3 H) 1.24 (br. s., 2 H) 1.50 - 1.71 (m, 6 H) 1.78 - 1.96 (m, 2 H) 1.97 - 2.13 (m, 2 H) 2.55 - 2.65 (m, 2 H) 2.67 - 2.88 (m, 6 H) 2.90 - 3.04 (m, 3 H) 3.11 (d, *J*=12.96 Hz, 1 H) 3.19 - 3.32 (m, 2 H) 3.37 (br. s., 1 H) 3.52 (dd, *J*=13.08, 3.30 Hz, 2 H) 3.65 (d, *J*=10.03 Hz, 2 H) 3.70 - 3.94 (m, 2 H) 6.73 (m, *J*=8.07 Hz, 2 H) 7.01 (m, *J*=8.07 Hz, 2 H) 7.15 - 7.31 (m, 9 H) 8.50 (d, *J*=5.01 Hz, 1 H) **m/z** calcd $C_{44}H_{55}N_5O_5$ $[M+H]^+$ 734.42, found 734.15 (MALDI), 734.15 (MS ESI) **Purity** LC-MS: 99.0% (254 nm, peak height).

**N-(4-((S)-1-(((S)-1-((R)-3-cyclohexyl-2-((S)-5-(4-hydroxybenzyl)-2,3-dioxo-4-phenethylpiperazin-1-yl)propyl)pyrrolidin-2-yl)methyl)-5,6-dioxopiperazin-2-yl)butyl)-5-((3aS,4S,6aR)-2-oxohexahydro-1H-thieno[3,4-d]imidazol-4-yl)pentanamide.** Using Scheme 3 for the synthesis of biotin tagged pyrrollidine-bis-diketopiperazines compound 2529-3 was synthesized using the following reagents: (100 mg) MBHA resin starting material, Fmoc-L-Lysine(Boc) -OH ($R_1$), Fmoc-D-Cyclohexylalanine-OH ($R_2$), Fmoc-L-Tyrosine(2-Br-Z)-OH ($R_3$), and Phenylacetic Acid ($R_4$). The final crude product was purified using HPLC as described above, with a gradient of (B) 0/5, 2/5, 4/20, 40/55. **Isolated Mass** 4.9mg **%yield** 4.33%.

**$^1$H NMR** (400 MHz, DMSO-$d_6$) δ ppm 9.63 (br. s., 1H) 9.35 (br. s., 1H) 8.56 (br. s., 1 H) 7.81 (br. s., 1 H) 7.29 (br. s., 1 H) 7.24 (br. s., 2 H) 7.01 (br. s., 1 H) 6.72 (br. s., 1 H) 6.40 (d, J=15.89 Hz, 1 H) 4.90 (br. s., 1 H) 4.31 (br. s., 1 H) 3.93 - 4.17 (m, 1 H) 3.63 - 3.87 (m, 4 H) 3.60 (br. s., 1 H) 3.53 (br. s., 2 H) 3.44 (br. s., 14 H) 3.31 (br. s., 2 H) 3.20 (br. s., 2 H) 3.09 (br. s., 1 H) 3.04 (br. s., 1 H) 2.83 (d, J=11.49 Hz, 3 H) 2.74 (br. s., 1 H) 2.59 (d, J=12.72 Hz, 4 H) 2.28 (br. s., 1 H) 2.04 (br. s., 3 H) 1.89 (br. s., 1 H) 1.81 (d, J=9.41 Hz, 1 H) 1.69 (br. s., 2 H) 1.63 (br. s., 3 H) 1.48 (br. s., 2 H) 1.42 (br. s., 2 H) 1.29 (br. s., 4 H) 1.19 (br. s., 2 H) 0.96 (br. s., 2 H) **m/z** calcd $C_{51}H_{72}N_8O_7S$ $[M+H]^+$ 941.53, found 471.15 (MS ESI) **Purity** LC-MS: 99.0% (254 nm, peak height).

**N-((S)-6-((S)-2-(((S)-6-benzyl-2,3-dioxopiperazin-1-yl)methyl)pyrrolidin-1-yl)-5-((S)-5-(4-hydroxybenzyl)-2,3-dioxo-4-phenethylpiperazin-1-yl)hexyl)-5-((3aS,4S,6aR)-2-oxohexahydro-1H-thieno[3,4-d]imidazol-4-yl)pentanamide.** Using Scheme 3 for the synthesis of Biotin tagged pyrrollidine-bis-diketopiperazines compound 2529-5 was synthesized using the following reagents: (100mg) MBHA resin starting material, Fmoc-L-Phenylalanine -OH ($R_1$), Fmoc-L-Lysine(Boc) -OH ($R_2$), Fmoc-L-Tyrosine(2-Br-Z)-OH ($R_3$), and Phenylacetic Acid ($R_4$). The final crude product was purified using HPLC as described above, with a gradient of (B) 0/5, 2/5, 4/20, 40/55. **Isolated Mass** 6.4 mg **%yield** 5.70%.

$^{1}$**H NMR** (400 MHz, DMSO-*d*$_6$) δ ppm 9.53 (br. s., 1 H) 9.35 (br. s., 1 H) 8.62 (br. s., 1 H) 7.82 (br. s., 1 H) 7.25 - 7.35 (m, 6 H) 7.22 (br. s., 1 H) 7.01 (br. s., 2 H) 6.72 (br. s., 1 H) 6.30 - 6.46 (m, 1 H) 4.76 (br. s., 1 H) 4.30 (br. s., 1 H) 4.12 (br. s., 1 H) 4.02 (d, J=12.47 Hz, 1 H) 3.78 (br. s., 2 H) 3.56 - 3.69 (m, 4 H) 3.51 (br. s., 10 H) 3.33 (br. s., 2 H) 3.16 (d, J=11.62 Hz, 2 H) 2.92 - 3.11 (m, 4 H) 2.76 - 2.92 (m, 4 H) 2.70 (br. s., 1 H) 2.57 (d, J=12.10 Hz, 1 H) 2.26 (br. s., 1 H) 2.02 (br. s., 3 H) 1.84 (br. s., 1 H) 1.59 (br. s., 1 H) 1.43 (br. s., 5 H) 1.29 (br. s., 2 H) **m/z** calcd $C_{51}H_{66}N_8O_7S$ $[M+H]^+$ 935.48, found 468.45 (MS ESI) **Purity** LC-MS: 99.0% (254 nm, peak height).

**N-(4-((S)-4-((R)-1-((S)-2-(((S)-6-benzyl-2,3-dioxopiperazin-1-yl)methyl)pyrrolidin-1-yl)-3-cyclohexylpropan-2-yl)-5,6-dioxo-1-phenethylpiperazin-2-yl)butyl)-5-((3aR,4R,6aS)-2-oxohexahydro-1H-thieno[3,4-d]imidazol-4-yl)pentanamide.** Using Scheme 3 for the synthesis of Biotin tagged pyrrollidine-bis-diketopiperazines compound 2529-7 was synthesized using the following reagents: (100mg) MBHA resin starting material, Fmoc-L-Phenylalanine -OH ($R_1$), Fmoc-D-Cyclohexylalanine-OH ($R_2$), Fmoc-L-Lysine(Boc) -OH ($R_3$), and Phenylacetic Acid ($R_4$). The final crude product was purified using HPLC as described above, with a gradient of (B) 0/5, 2/5, 4/20, 40/55. **Isolated Mass** 16.1mg **%yield** 14.50%.

$^{1}$**H NMR** (400 MHz, DMSO-*d*$_6$) δ ppm 9.39 (br. s., 1 H) 8.65 (br. s., 1 H) 7.78 (br. s., 1 H) 7.33 (br. s., 5 H) 7.28 (br. s., 3 H) 6.29 - 6.47 (m, 1 H) 4.86 (br. s., 1 H) 4.21 - 4.36 (m, 1 H) 4.12 (br. s., 1 H) 4.03 (d, J=12.72 Hz, 2 H) 3.79 (br. s., 4 H) 3.59 - 3.75 (m, 7 H) 3.52 (d, J=18.58 Hz, 3 H)3.25 - 3.43 (m, 2 H) 3.18 (br. s., 2 H) 2.97 - 3.10 (m, 4 H) 2.92 (br. s., 2 H) 2.70 - 2.87 (m, 1 H) 2.57 (d, J=11.62 Hz, 1 H) 2.29 (br. s., 1 H) 2.04 (br. s., 2 H) 1.99 (br. s., 1 H) 1.85 (br. s., 2 H) 1.65 (br. s., 2 H) 1.60 (br. s., 3 H) 1.48 (br. s., 2 H) 1.33 - 1.44 (m, 3 H) 1.29 (br. s., 4 H) 1.13 (br. s., 3 H) 0.72 - 0.96 (m, 2 H) **m/z** calcd $C_{51}H_{72}N_8O_6S$ $[M+H]^+$ 925.53, found 463.20 (MS ESI) **Purity** LC-MS: 95.0% (254 nm, peak height).

**Supplemental Table 1. Viability testing of biotinylated analogs of compound 2155-14 with WM266-4 cells.** All units are $IC_{50}$, µM (n=3). Numbers in 2155-14 structure indicate positions of substitutions of basic scaffold.

| ID | Structure | R1 | R2 | R3 | R4 | $IC_{50}$, µM |
|---|---|---|---|---|---|---|
| 2155-14/2529-1 | 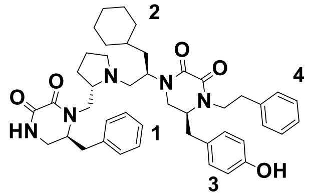  | | | | | 4.0/1.6 |
| 2529-3 | 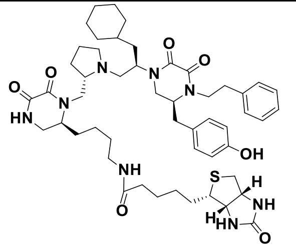 | Biotin | | | | >100 |
| 2529-5 | 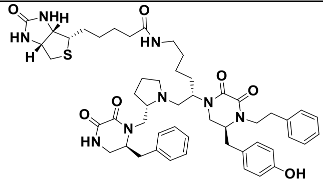 | | Biotin | | | >100 |
| 2529-7 | 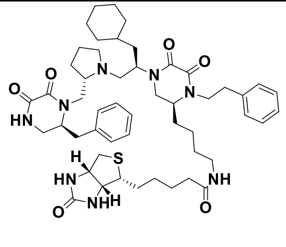 | | | Biotin | | 3.3 |

**Supplementary Figure 2. Comparison of sequence coverage of bands 3 and 4 from pulldown experiment.** Yellow = identified peptides, green = modified amino acids. Top = coverage of band 4, bottom = coverage of band 3. Please note that there is a difference in coverage of amino acids 3-14 between band 3 and 4 suggesting that band 3 is hnRNP B1 and band 4 is hnRNP A2.

**P22626 (100%), 37,430.3 Da**
**Heterogeneous nuclear ribonucleoproteins A2/B1 n=12 Tax=Boreoeutheria RepID=ROA2_HUMAN**
**22 exclusive unique peptides, 34 exclusive unique spectra, 89 total spectra, 189/353 amino acids (54% coverage)**

```
MEKTLETVPL ERKKREKEQF RKLFIGGLSF ETTEESLRNY YEQWGKLTDC
VVMRDPASKR SRGFGFVTFS SMAEVDAAMA ARPHSIDGRV VEPKRAVARE
ESGKPGAHVT VKKLFVGGIK EDTEEHHLRD YFEEYGKIDT IEIITDRQSG
KKRGFGFVTF DDHDPVDKIV LQKYHTINGH NAEVRKALSR QEMQEVQSSR
SGRGGNFGFG DSRGGGGNFG PGPGSNFRGG SDGYGSGRGF GDGYNGYGGG
PGGGNFGGSP GYGGGRGGYG GGGPGYGNQG GGYGGGYDNY GGGNYGSGNY
NDFGNYNQQP SNYGPMKSGN FGGSRNMGGP YGGGNYGPGG SGGSGGYGGR
SRY
```

**P22626 (100%), 37,430.3 Da**
**Heterogeneous nuclear ribonucleoproteins A2/B1 n=12 Tax=Boreoeutheria RepID=ROA2_HUMAN**
**23 exclusive unique peptides, 30 exclusive unique spectra, 58 total spectra, 219/353 amino acids (62% coverage)**

```
MEKTLETVPL ERKKREKEQF RKLFIGGLSF ETTEESLRNY YEQWGKLTDC
VVMRDPASKR SRGFGFVTFS SMAEVDAAMA ARPHSIDGRV VEPKRAVARE
ESGKPGAHVT VKKLFVGGIK EDTEEHHLRD YFEEYGKIDT IEIITDRQSG
KKRGFGFVTF DDHDPVDKIV LQKYHTINGH NAEVRKALSR QEMQEVQSSR
SGRGGNFGFG DSRGGGGNFG PGPGSNFRGG SDGYGSGRGF GDGYNGYGGG
PGGGNFGGSP GYGGGRGGYG GGGPGYGNQG GGYGGGYDNY GGGNYGSGNY
NDFGNYNQQP SNYGPMKSGN FGGSRNMGGP YGGGNYGPGG SGGSGGYGGR
SRY
```

**Supplementary Figure 3. Optimization of DDX1, hnRNP H2, and hnRNP A2/B1 siRNA knock-down conditions.** Encircled concentrations of respective siRNAs were chosen for downstream experiments. C1 = scrambled siRNA control at 100nM.

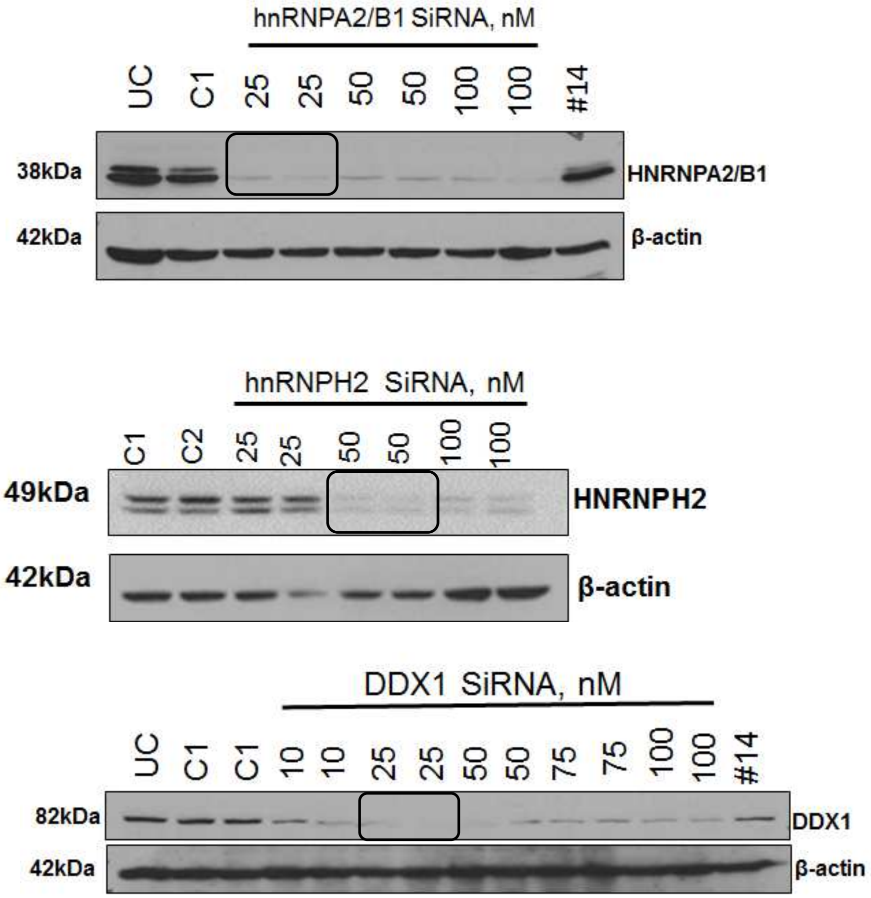

Supplement: Supplemental Material [file NIHMS1067973-supplement-Supplemental_Material.pdf]
